# Mesoporous Sn-In-MCM-41 Catalysts for the Selective Sugar Conversion to Methyl Lactate and Comparative Life Cycle Assessment with the Biochemical Process

**DOI:** 10.1021/acssuschemeng.1c04655

**Published:** 2022-02-21

**Authors:** Óscar de la Iglesia, Miryan Sarango, Mikel Munárriz, Magdalena Malankowska, Alberto Navajas, Luis M. Gandía, Joaquín Coronas, Carlos Téllez

**Affiliations:** † Centro Universitario de la Defensa Zaragoza, Academia General Militar, 50090 Zaragoza, Spain; ‡ Instituto de Nanociencia y Materiales de Aragón (INMA), CSIC-Universidad de Zaragoza, 50018 Zaragoza, Spain; § Department of Chemical and Environmental Engineering, Universidad de Zaragoza, 50018 Zaragoza, Spain; ∥ Department of Science, Universidad Pública de Navarra, Campus de Arrosadia, 31006 Pamplona, Spain; ⊥ Institute for Advanced Materials and Mathematics (InaMat2), Universidad Pública de Navarra, Edificio Jerónimo de Ayanz, Campus de Arrosadia, 31006 Pamplona, Spain

**Keywords:** heterogeneous catalysis, sugar conversion, lactic acid, Sn-In-MCM-41, mesoporous materials, life cycle assessment

## Abstract

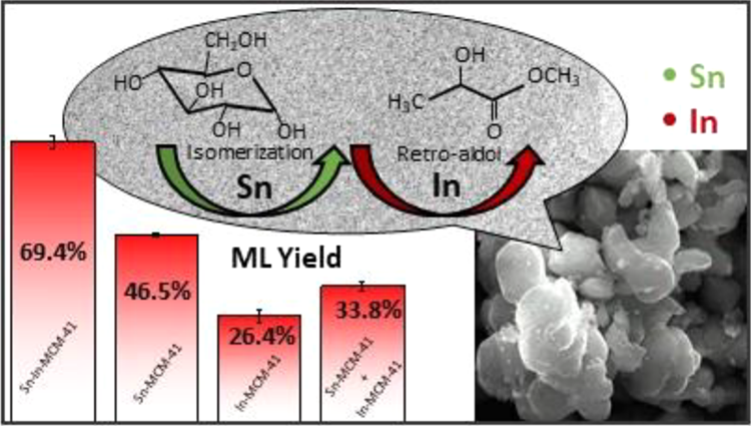

The use of biomass for the production of energy and higher added value products is a topic of increasing interest in line with growing environmental concerns and circular economy. Mesoporous material Sn-In-MCM-41 was synthesized for the first time and used as a catalyst for the transformation of sugars to methyl lactate (ML). This catalyst was characterized in depth by various techniques and compared with Sn-MCM-41 and In-MCM-41 catalysts. In the new Sn-In-MCM-41 material, both metals, homogeneously distributed throughout the mesoporous structure of MCM-41, actuate in a cooperative way in the different steps of the reaction mechanism. As a result, yields to ML of 69.4 and 73.9% in the transformation of glucose and sucrose were respectively reached. In the case of glucose, the ML yield 1.5 and 2.6 times higher than those of Sn-MCM-41 and In-MCM-41 catalysts, respectively. The Sn-In-MCM-41 catalyst was reused in the transformation of glucose up to four cycles without significant loss of catalytic activity. Finally, life cycle assessment comparison between chemical and biochemical routes to produce ML allowed us to conclude that the use of Sn-In-MCM-41 reduces the environmental impacts compared to Sn-MCM-41. Nevertheless, to make the chemical route comparable to the biochemical one, improvements in the catalyst and ML synthesis have to be achieved.

## Abstract 2

Tin- and indium-based mesoporous silica MCM-41 improves the chemical route to produce ML. The increase of ML yield improves the chemical route to overcome the biochemical route.

## Introduction

Lactic acid is a platform chemical that can be used for a wide range of applications in the chemical, pharmaceutical, cosmetics, and food industries,^1^ as well as for the production of poly-lactic acid, which is a biodegradable, biocompatible, and environmentally friendly biopolymer.^2^ Lactic acid is obtained industrially mainly through the fermentation of carbohydrates (usually pentoses or hexoses) in aqueous medium. Nevertheless, this process has some drawbacks related to nutrient costs, generation of gypsum waste in the neutralization step, and low volumetric productivities.^3^

In the last years, advances in the catalytic conversion of carbohydrates to lactic acid and its derivatives to overcome the drawbacks of fermentation have been made.^3,4^ The first development was the conversion of trioses with homogeneous catalysts, which requires a Lewis and/or Brønsted acid catalyst.^3,4^ Hayashi and Sasaki^5^ pioneered the conversion of trioses with alcohols to obtain alkyl lactates using homogeneous Lewis acidic tin halides. In their study, tin halides exhibited higher catalytic activity than other metal compounds tested. Since then, other homogeneous catalysts have been reported for the production of alkyl lactates, such as NaOH-neutralized SnCl_4_^6^ or Sn^4+^-based organometallic complexes.^7^

Purification of lactic acid from aqueous solutions is a complex process which involves the esterification of lactic acid with an alcohol to obtain the corresponding alkyl lactate, distillation, and subsequent hydrolysis, which produce high energy cost.^8^ Therefore, carrying out the reaction in an alcoholic medium facilitates the purification process of the desired product and decreases its cost.

From the green and sustainable chemistry point of view, heterogeneous acid catalysts are preferable over homogeneous catalysts, owing to easy handling, simple workup, and recyclability. Different Sn-containing heterogeneous catalysts based on zeolites, ordered mesoporous silicas, or mixed oxides have been used in the conversion of trioses to lactic acid or alkyl lactates due to their high catalytic activity for this reaction, for instance, Sn-MFI-type zeolite,^9^ Sn-MCM-41,^9−12^ Sn-SBA-15,^9^ Sn-MWW-type zeolite,^13^ Sn-Si mixed oxides,^14^ or Sn-Nb mixed oxides.^15^ Further, other metal oxides without tin (e.g., γ-Al_2_O_3_^16^ or Nb_2_O_5_^17^) or metal phosphates (e.g., with Sn^18^ or Nb^19^) have been tested for the conversion of dihydroxyacetone to lactic acid derivatives. Life cycle assessment (LCA) has been used to evaluate the environmental impacts of methyl lactate (ML) production by Lewis acid-catalyzed isomerization of dihydroxyacetone.^20^

Another way for the production of lactic acid or alkyl lactates is the use of glucose, a product of the hydrolysis of polysaccharides such as starch and cellulose, or even sucrose, which is closer to the reality of an industrially viable process.^21^ This route involves isomerization, retro-aldol, and hydrolysis (only for sucrose) reactions, which need Lewis and/or Brønsted acid catalysts^3,4^ as for trioses. The main difference is that higher temperatures are required (≥150 °C) than in the case of trioses (usually tested at about 90 °C), which causes more side reactions, and consequently lower yields are obtained.^3^

In this context, Holm et al.^22^ reported the conversion of sucrose to alkyl lactates using zeolites Ti-beta, Zr-beta, and Sn-beta with a maximum yield of ML of 64% at 160 °C and 20 h. After that work, other authors reported the utilization of several heterogeneous catalysts: carbon–silica composite,^10^ Sn-MWW-type zeolite,^13^ Sn-MCM-41,^23,24^ Mg-MOF-74,^25^ Sn-beta zeolite with different modifications,^24,26−29^ γ-NiOOH,^30^ or NiO.^31^

In 2016, Nemoto et al.^32^ studied the activity of different metal chlorides as homogeneous catalysts in the transformation of sugars to ML. They reported that indium chloride had higher activity than tin chloride for the transformation of fructose, but lower activity if the sugar was dihydroxyacetone; as a result, the authors claimed that indium catalyzes the retro-aldol reaction and tin catalyzes the isomerization. Next, they applied a combined catalyst with both indium and tin chlorides to the conversion of fructose and reached a maximum yield of 70% of ML for In/(In + Sn) atomic ratios from 0.5 to 0.83 at 160 °C and 10 h. Recently, the same group reported the use of the same catalysts for the conversion of cellulose to ML.^33^ In 2018, Deng et al.^34^ examined different cation combinations for the catalytic conversion of several carbohydrates. They reached a yield to lactic acid of 80% at 180 °C for 2 h with the combination of Al(III) and Sn(II) salts. This catalyst showed better performance than other single and dual cationic combinations used.

Regarding heterogeneous catalysts, Fe-doped SnO_2_^35^ and Ti/Sn bimetallic MIP-177-LT^36^ have been also used for the conversion of carbohydrates to ML. Xia et al.^37^ used an In-Sn-beta zeolite for the transformation of glucose to lactic acid. Their highest yield to ML was 53% at 190 °C and 2 h with an In/(In + Sn) atomic ratio of 0.7. Recently, with hierarchical bimetal-doped Beta zeolite catalysts, other authors have achieved ML yields of ca. 67% in the transformation of glucose with Zn–Sn^38^ or Fe–Sn^39^ at 200 °C. Therefore, it can be seen that either for homogeneous or for heterogeneous catalysts, the combination of two metals has a synergistic effect that improves the performance of the catalyst for this specific reaction.

In previous studies, we reported the catalytic transformation of sugars to ML with promising results using different catalysts: mesoporous Sn-MCM-41,^23^ metal–organic frameworks as Zn imidazolate ZIF-8,^40^ Sn-based carboxylates UZAR-S10 and MIP-177-LT^36^ (this being a Ti/Sn bimetallic catalyst), and exfoliated layered stannosilicate UZAR-S4.^41^

Considering that Sn-MCM-41 reached a yield to ML of 43% from glucose, with no significant loss of catalytic activity for three catalytic cycles^23^ and the high catalytic activity exhibited by the combination of tin and indium in other catalytic materials,^32,37^ in this work, we carried out the synthesis of ordered mesoporous silica material Sn-In-MCM-41 and used it for the sugar transformation.

The preparation of Sn-MCM-41 and its use in catalytic reactions has been reported on different occasions.^12,23,24,42^ Nevertheless, indium has hardly been used as an active component of a heterogeneous catalyst in the production of ML, except in a recent work where the In/γ-Al_2_O_3_ catalyst^43^ was applied in the conversion of glucose to ML reaching a yield of 40% at 180 °C for 10 h. On the other hand, publications about the synthesis of In-MCM-41 are scarce;^44,45^ in fact, an indium-modified Al-MCM-41 was used as a catalyst for the Knoevenagel reaction.^46^ To the best of our knowledge, this is the first time that an ordered mesoporous silica with tin and indium substituted in its framework is applied to this kind of reactions, achieving a high performance in terms of ML yield. Finally, an LCA comparison between chemical and biochemical routes has been carried out to evaluate their environmental impacts.

## Materials and Methods

### Synthesis of Catalysts

Sn-In-MCM-41 was prepared by dissolving 4.06 g of hexadecyltrimethyl-ammonium bromide (CTABr) (98%, Sigma-Aldrich) and 1.67 g of NaOH (98%, Scharlau) in 180 mL of deionized water. Next, 0.10 g of SnCl_2_·2H_2_O (98%, Sigma-Aldrich) and 0.098 g of InCl_3_ (98%, Sigma-Aldrich) were added to this solution, and finally 13.6 g of tetraethylorthosilicate (TEOS) (98%, Sigma-Aldrich) was added as well. All these components were mixed at room temperature. The molar composition of this gel was 6 TEOS: 1 CTABr: 900 H_2_O: 4 NaOH: 0.04 SnCl_2_: 0.04 InCl_3_. The mixture was heated at 80 °C for 8 h under reflux. The solid product was recovered by filtration, washed with deionized water, and dried at 70 °C overnight. After drying, Sn-In-MCM-41 was calcined in static air at 650 °C for 8 h. In each synthesis, a Sn-In-MCM-41 amount of 3.2–3.3 g was obtained. For comparison with Sn-In-MCM-41, Sn-MCM-41 and In-MCM-41 were also synthetized with the same Si/metal atomic ratio and applied to the same reaction. As a metal source, 0.20 g of SnCl_2_·2H_2_O was added for Sn-MCM-41 and 0.196 g of InCl_3_ was added in the case of In-MCM-41.

### Catalyst Characterization

Phase identification was acquired by a D-Max 2500 Rigaku X-ray diffractometer with a copper anode and a graphite monochromator using Cu-Kα_1_ radiation (λ = 1.5418 Å), taking data from 2θ = 1° to 10° at a scan rate of 0.03°·s^–1^ and operating parameters of 40 kV and 80 mA. Elemental analysis was performed to determine the Si/Sn and Si/In ratios in MCM-41 samples using a Thermo Electron ARL ADVATXP X-ray fluorescence (XRF) sequential spectrometer equipped with an X-ray tube equipped with a Be window and Rh anode.

The total amount of Brønsted acid sites was determined by titration with NaOH. In a typical analysis, 50 mg of the catalyst was dispersed in 100 mL of deionized water and three aliquots of this sample were analyzed. The titration was carried out with a 2·10^–4^ M solution of NaOH (97%, Sigma-Aldrich), whose concentration was previously determined using potassium hydrogen phthalate (PanReac). In all cases, phenolphthalein (PanReac) was used as an indicator.

Pyridine Fourier transform infrared (FTIR) was used to determine the type of Brønsted and Lewis acidic sites. Catalysts, previously dried at 100 °C overnight in an oven, were exposed for adsorption to the pyridine (PanReac) vapor for 24 h at ambient temperature. After this adsorption step, catalysts were treated separately with a nitrogen stream of 50 mL(STP)·min^–1^ at 80 °C for 30 min to remove the physisorbed pyridine. FTIR spectra were measured with a Vertex 70 of Bruker and a Specac’s Golden Gate ATR. Baseline correction was carried out, and FTIR spectra were provided as achieved.

Nitrogen adsorption–desorption isotherms were obtained with a Micromeritics Tristar 3000 at 77 K. Before these measurements, the samples were degassed at 200 °C during 8 h under vacuum. The specific surface area was calculated using the Brunauer–Emmett–Teller (BET) equation. Pore diameters were determined by the 4 V/A method from BET data, by the Barrett–Joyner–Halenda (BJH) method, and by the density functional theory (DFT) method.

Scanning electron microscopy (SEM) and energy-dispersive X-ray spectroscopy (EDX) were performed using an Inspect F50 model microscope (FEI) operated at 20 kV. The samples were prepared over a carbontape and coated with 20 nm of carbon under vacuum conditions. Morphology of particles was observed by transmission electron microscopy (TEM). The images were taken with an FEI TECNAI F30 at 80–300 kV. Thermal behavior was determined by thermogravimetric analyses (TGA), which were carried out using a Mettler Toledo TGA/SDTA 851^e^ system. Samples (about 5 mg) were placed in 70 μL alumina pans and heated under air flow up to 800 °C at a heating rate of 10 °C·min^–1^.

X-ray photoelectron spectroscopy (XPS) was used to analyze the binding energy values and the atomic surface concentration of tin and indium. The XPS analyses were performed with an Axis Ultra DLD (Kratos Tech.). The spectra were excited by a monochromatized Al Kα source (1486.6 eV) at 15 kV and 10 mA, and a pass energy of 20 eV was used for the individual peak regions. The effects of the sample charging were eliminated by correcting the observed spectra for a C 1 s binding energy value of 284.9 eV.

Temperature-programmed reduction (TPR) experiments were performed with H_2_ in a Quantachrome ChemBET Pulsar automatic chemisorption analyzer. In a typical run, 100 mg of the catalyst was placed in a quartz microreactor. The sample was pretreated at 200 °C for 15 h under Ar flow (20 mL(STP)·min^–1^), and TPR profiles were recorded in a flow of H_2_ (5 v/v%) in Ar (total flow 20 mL(STP)·min^–1^) and a heating rate of 10 °C·min^–1^ up to 800 °C.

### Catalytic Tests

Conversion of sugars to ML was carried out in a 35 mL Teflon autoclave. For the catalytic tests with glucose, 225 mg of d-(+)-glucose (99%, Alfa Aesar), 8.0 g of methanol (Multisolvent HPLC grade, Scharlau), 160 mg of the catalyst, and 30 mg of naphthalene (99%, Sigma-Aldrich), as internal standard, were added to the autoclave. The reaction was performed at 160 °C for 20 h in a rotary oven at 15 rpm. With this rotation speed, no mass transfer limitations were observed.^40^ The procedure was the same for the experiments performed with sucrose (99%, Sigma-Aldrich), with the only difference being the time of the experiment equal to 24 h because for sucrose, the yield of ML still increases after 20 h, but for glucose, it remains constant.^40^ For the catalytic cycles, when the reaction was over, the catalyst was recovered by centrifugation and dried in an oven at 70 °C overnight prior to its reutilization.

The determination of products in the reaction liquid was carried out in the gas chromatograph (Agilent 6850) coupled with an Agilent 5975C mass spectrometry detector, as has already been described elsewhere.^40^ Sugars were determined using a commercial analytical method (Sucrose/Fructose/d-Glucose Assay Kit, Megazyme). The absorbance was measured using a V-670 Jasco UV–vis spectrophotometer.^40^ This enzymatic kit is able to detect the sugars even if they have some substituent.

### Life Cycle Assessment

LCA allows for the quantitative analysis of the material and energy efficiency of a process, the identification of environmental hazards, as well as the establishment of a reference to compare different ways to obtain the same product. The LCA presented in this work follows the recommendations given by the European Platform on LCA.^47^ The software GaBi 9.5 Pro. was used for the LCA simulation. The functional unit of this study is the production of 1 kg of ML by biochemical and chemical (Sn-MCM-41 and Sn-In-MCM-41 catalysts) routes. Databases associated with GaBi 9.5 Pro. Software have also been used for the study. When possible, processes located in Spain were considered; otherwise data from the European Union or Germany were applied. Inputs to calculate the different impacts are summarized in Table 1. Furthermore, an explanation of how each input on LCA software was simulated is given in Supporting Information.

**Table 1:** Life Cycle Inventory of the Biochemical and Chemical (Sn-MCM-41 and Sn-In-MCM-41 Catalysts) Routes and of the Improved Conditions for the Production of 1 kg of ML

	biochemical route	Sn-MCM-41	Sn-In-MCM-41	Sn-In-MCM-41 improved conditions	Sn-In-MCM-41 improved conditions and 100% ML yield
bacteria [kg]	0.009				
nutrients [kg]	0.061				
glucose [kg]	1.30	2.02	1.39	1.39	0.86
methanol [kg]	0.017	3.57^a^	2.47^a^	1.23^a^	0.76^a^
H_2_SO_4_ [kg]	1.30				
thermal energy (MJ)	107	97	72.5	37.2	21.1
water [kg]	106	19.8	13.7	13.7	8.5
Ca(OH)_2_ [kg]	0.69				
SnCl_2_ [kg]		0.021	8.00·10^–3^	8.00·10^–3^	4.99·10^–3^
InCl_3_ [kg]			7.50·10^–3^	7.50·10^–3^	4.66·10^–3^
TEOS [kg]		1.51	1.04		
Na_2_SiO_3_ [kg]				0.61	0.38
CTABr [kg]		0.44	0.31	0.31	0.19
NaOH [kg]		0.17	0.12	0.12	0.08

a 5% of the total required. 95% is recovered by distillation. Methanol required for glucose conversion to ML is provided by this 5%.

The calculations lead to the achievement of 16 environmental impact indicators (EIIs), which allows for the comparison between the different processes analyzed. Table S2 presents the EIIs used together with their corresponding units and their recommendation level (level I: recommended and satisfactory; level II, recommended but in need of some improvements; or level III, recommended, but to be applied with caution).

## Results and Discussion

### Catalyst Characterization

TGA of the as-synthesized and calcined Sn-In-MCM-41 were carried out. As can be seen in Figure S1, the as-synthesized MCM-41 sample shows several weight losses usually found in literature.^48^ Weight loss below 130 °C is related to the presence of physisorbed water in the surface or inside the pores of the MCM-41, losses between 130–325 °C are mainly caused by the degradation of the surfactant, and losses above 300 °C correspond to the oxidation of carbonaceous species and the removal of water during the condensation of silanol groups. The main weight loss in the as-synthesized sample is due to the presence of the surfactant that acts as the organic structure-directing agent (OSDA) to obtain the MCM-41 structure. The weight loss in the calcined sample was negligible. This result ensures that the calcination method was proper in order to completely remove the OSDA from the pores.

Low-angle X-ray diffraction patterns of the as-synthesized and calcined Sn-In-MCM-41 are shown in Figure S2. The sample presents two peaks at 2θ angles of 2.2° and 3.8°, which corresponds to (100) and (110) plane reflections, typical of an MCM-41 mesoporous ordered structure. After calcination, Sn-In-MCM-41 maintained the structure but with some shrinking, and the intensity corresponding to the (100) plane displaced to a 2θ angle of 3.5°. The d-spacing for (100) planes was calculated using the Bragg’s law. d_100_ varied from 4.0 nm in the as-synthesized sample to 2.5 nm in the calcined one, indicating a contraction of the structure during the calcination. The low-angle X-ray diffractogram of In-MCM-41 (see Figure S3) is similar to that of Sn-In-MCM-41, where a contraction in the structure during calcination from a value of d_100_ 3.6 to 2.8 nm occurred as well. In the case of Sn-MCM-41, the X-ray diffractogram is similar to that reported previously^23^ and in agreement with a contraction in the structure with a variation of d_100_ from 4.0 to 2.6 nm, similar to that of Sn-In-MCM-41 (see Table 2).

**Table 2:** Textural Properties and Metal Content Determined by X-ray Fluorescence and X-ray Photoelectron Spectroscopy for the Prepared Catalysts and Brønsted Acid Sites Determined by Titration of the Prepared Catalysts

	Sn-In-MCM-41	Sn-MCM-41	In-MCM-41
BET surface area [m^2^·g^–1^]	899 ± 15	1034 ± 14	978 ± 25
pore volume^a^ [cm^3^·g^–1^]	0.42	0.48	0.48
pore diameter (4 V/A)^b^ [nm]	1.9	1.9	2.0
pore diameter (BJH) [nm]	2.0	2.1	2.3
pore diameter (DFT) [nm]	1.8	1.8	2.1
d_100_ as synthesized [Å]	40	40	36
d_100_ calcined [Å]	25	26	28
primary mesopore volume^c^ [cm^3^·g^–1^]	0.29	0.34	0.34
pore diameter^c^ [nm]	1.9	2.0	2.2
Sn XRF (wt %)	1.88 ± 0.04	4.71 ± 0.09	
In XRF (wt %)	1.22 ± 0.02		2.49 ± 0.08
atomic Si/Sn gel	150	75	
atomic Si/In gel	150		75
In/(In + Sn) gel	0.50	0	1
atomic Si/Sn XRF	101	40	
atomic Si/In XRF	151		74
In/(In + Sn) XRF	0.40	0	1
atomic Si/Sn XPS	52	28	
atomic Si/In XPS	23		22
In/(In + Sn) XPS	0.69		
Brønsted acid sites [mmol·g^–1^]	0.117 ± 0.002	0.123 ± 0.002	0.128 ± 0.002
mmol ac/mmol metal	0.44	0.31	0.59

a At *P*/*P*_0_ = 0.97.

b From BET data.

c From Kruk et al.^54^

The amount of metal in Sn-In-MCM-41, Sn-MCM-41, and In-MCM-41 was determined by XRF (see Table 2). Because an X-ray beam can penetrate up to 20 μm as an average and the particles have a maximum size of less than 9 μm (see Figure 2), this technique gives the composition in the bulk. Sn-MCM-41 has a 4.7 wt % of tin, this value is within the experimental variation and it is slightly higher than that registered before (3.4 wt %).^23^ Sn-In-MCM-41 contains 1.9 wt % of tin and 1.2 wt % of indium. Attending to the possible experimental variation, the amount of tin should be in the range 1.7–2.4 wt % because we used half of the moles of tin in the synthesis gel; thus, it falls within the expected values. The Si/Sn and Si/In atomic ratios were 101 and 151, respectively. Because the Si/metal ratio in the gel was 150 for both metals, tin was incorporated to the material structure in greater proportion than silicon, while indium was incorporated in the same proportion. The same behavior was observed for Sn-MCM-41 and In-MCM-41, with an atomic ratio Si/Sn of 40 in Sn-MCM-41 (4.7 wt %), lower than the atomic ratio in the gel (Si/Sn = 75), while the atomic ratio in In-MCM-41 was 74 (2.5 wt %), similar to that in the gel (Si/In = 75). The incorporation of indium is more difficult, probably due to the fact that indium has different oxidation states, and when an In^3+^ cation substitutes the Si^4+^, a compensation cation is required to maintain the electroneutrality of the structure. Nevertheless, the substitution of silicon by tin is easier because both are able to act with an oxidation state +4. Furthermore, the ionic ratio of In^3+^ is larger than that of Sn^4+^ (0.81 vs 0.71 Å), which suggests a worse mobility during the synthesis of the indium-based materials, besides producing a greater distortion in the MCM-41 structure.

The Sn-In-MCM-41, Sn-MCM-41, and In-MCM-41 FTIR spectra in Figure S4 are typical for MCM-41 ordered mesoporous silicas: there is a wide band centered at 3350 cm^–1^ corresponding to hydrogen-bonded silanol groups and to adsorbed water. Another band at 1627 cm^–1^ is related to the adsorbed water. Three bands at 1045, 950, and 795 cm^–1^ indicate the presence of Si-O bonds that form the MCM-41 structure.^49^ The 950 cm^–1^ band is more intense in the case of Sn-MCM-41 followed by Sn-In-MCM-41, being less appreciated in sample In-MCM-41. This band has been assigned to the incorporation of heteroatoms in the structure of MCM-41,^50,51^ so that the substitution would be more feasible with tin than with indium as indicated above.

The number of Brønsted acid sites in Sn-In-MCM-41, Sn-MCM-41, and In-MCM-41 determined by titration is summarized in Table 2. In the three cases, there are similar concentrations of Brønsted acid sites, around 0.12–0.13 mmol·g^−1^ which confirm the presence of catalytic acid sites in the three catalysts. These values are similar to those previously published: Xu et al.^52^ reported a concentration of Brønsted acid sites in the range of 0.18 to 0.40 mmol·g^−1^ for Al-MCM-41, and Kim et al.^12^ reported 0.06 mmol·g^−1^ of Brønsted acid sites for Sn-MCM-41. Nevertheless, the catalysts containing indium have a higher ratio of Brønsted acid sites per metal unit (0.44, 0.31, and 0.59 mmol acid/mmol metal for Sn-In-MCM-41, Sn-MCM-41, and In-MCM-41, respectively). This is in agreement with the fact that the exchange of In^3+^ cations for Si^4+^ in the MCM-41 structure provides the insertion of a compensation proton, and as a consequence, acid sites are created. This effect has been reported when replacing Si^4+^ cations in tetrahedral positions by Al^3+^ cations.^53^ Furthermore, distortion of the structure caused by indium also provides the formation of silanol groups. Both situations lead to a higher Brønsted acidity.

**Figure 1:**
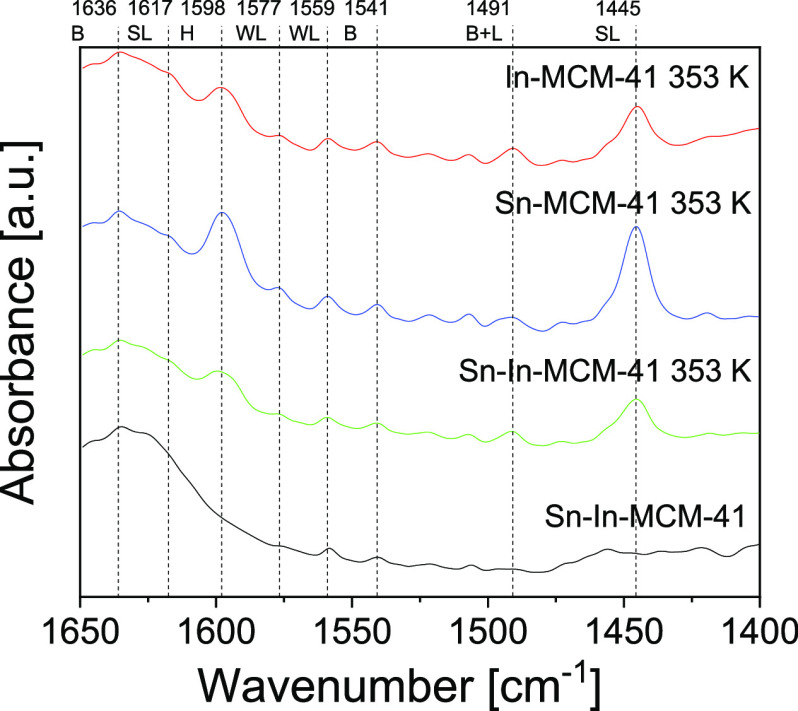
FTIR spectra of calcined Sn-In-MCM-41 and Sn-In-MCM-41, Sn-MCM-41, and In-MCM-41 after pyridine adsorption and heating at 353 K (B: Brønsted acid sites; L: Lewis acid sites; WL: weak Lewis acid sites; SL: strong Lewis acid sites; and H: hydrogen bond interactions). Figure S5 shows the spectra of Sn-MCM-41 and In-MCM-41 before pyridine adsorption.

In order to know the type of acidity of the prepared catalysts, pyridine FTIR analysis was performed. Figure 1 shows the FTIR spectra from calcined Sn-In-MCM-41 and from Sn-In-MCM-41, Sn-MCM-41, and In-MCM-41 after pyridine adsorption. After pyridine adsorption, several bands can be observed. Those at 1617 and 1445 cm^–1^ are due to strong Lewis acid sites, while absorbances at 1577 and 1559 cm^–1^ correspond to weak Lewis acid sites.^35^ There are two absorbances related to the Brønsted acid sites (B) at 1636 and 1541 cm^–1^.^55^ In addition, there is a band at 1598 cm^–1^ corresponding to the hydrogen bond interactions between pyridine and the catalyst surface (H)^35^ and another band centered at 1491 cm^–1^ due to the coordination of pyridine with Brønsted and Lewis acid sites (B + L).^55^ All the catalysts prepared possess Brønsted and Lewis acid sites, which make them suitable to catalyze retro-aldol and isomerization reactions for the production of ML. No significant differences can be observed in the absorption spectra, as the differences in the concentration of acid sites are low; only the absorbance at 1445 cm^–1^ is more intense for Sn-MCM-41, indicating that it has a higher amount of strong Lewis acids than the indium-containing catalyst. This effect already has been observed in beta zeolite catalysts.^37^

**Figure 2:**
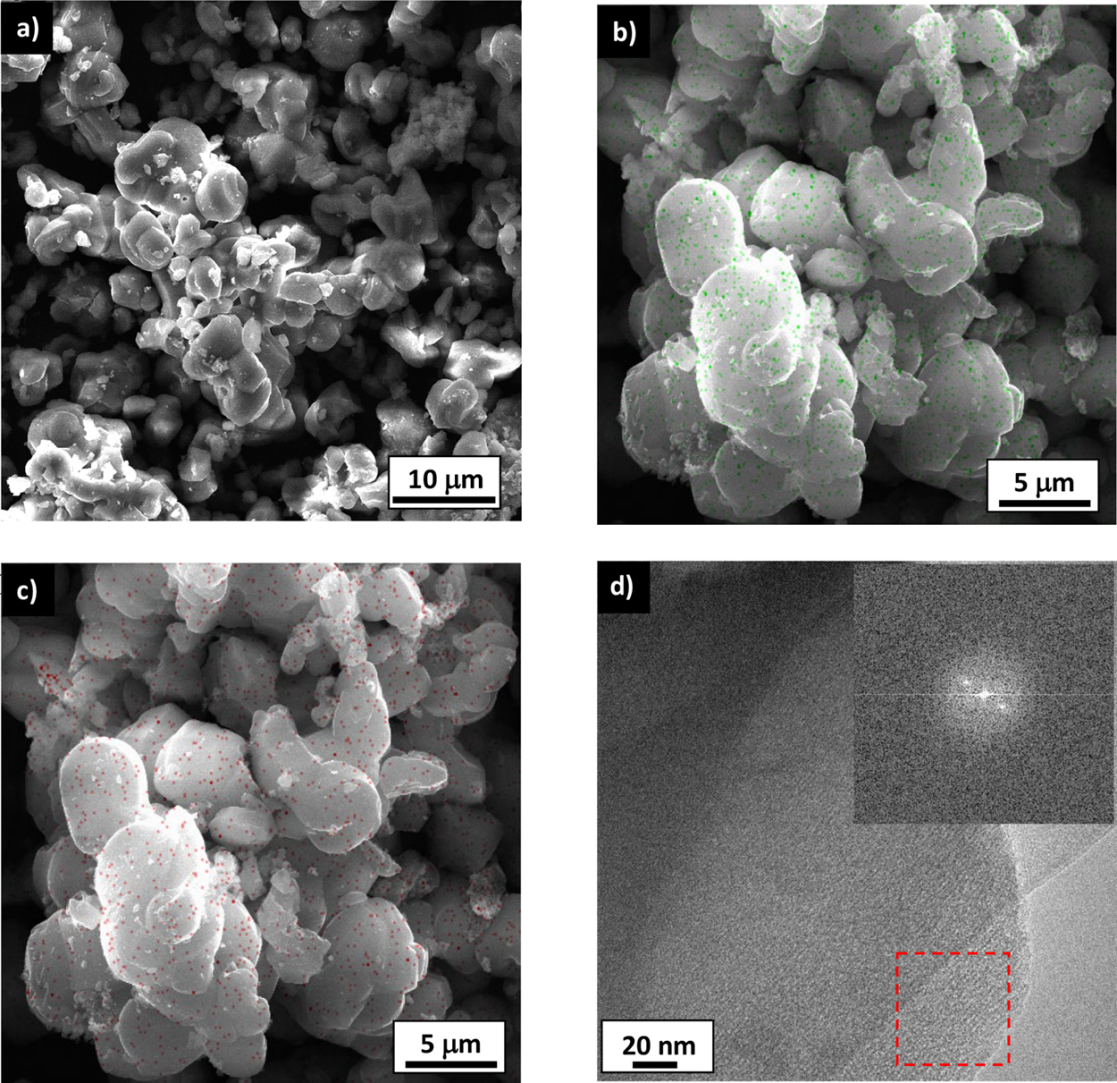
Electronic microscopy photographs of Sn-In-MCM-41: (a) SEM image of particles; (b) SEM image with EDX mapping of tin (green points); (c) SEM image with EDX mapping of indium (red points); and (d) TEM image of one particle. The red square indicates the selected area for the Fourier transform shown in the inset.

Figure 2a shows an SEM image of Sn-In-MCM-41 particles. A homogeneous particle size distribution can be observed, with a mean size of 5.8 ± 1.1 μm. Figure 2b,c corresponds to EDX mapping of tin and indium, respectively. There is a homogeneous distribution of both metals in the Sn-In-MCM-41 particles. From EDX, the concentration of tin and indium (2.0 ± 0.1 wt % and 1.1 ± 0.1 wt %, respectively) was also obtained. These values are similar to those obtained by XRF (Table 2).

Figure 2d shows a TEM image of calcined Sn-In-MCM-41. There are no evident black regions related to clusters of extraframework metals, which is in agreement with SEM–EDX images. Furthermore, the TEM image confirms that Sn-In-MCM-41 possesses an ordered porous structure. Fourier transform was another characterization method used to calculate the reciprocal space between planes. The inset in Figure 2d displays clear diffraction spots, confirming the crystalline structure of MCM-41 and its hexagonal shape, in concordance with the corresponding X-ray diffraction pattern (see Figure S2). Two aligned points can be observed in the reciprocal that correspond to parallel (100) planes. By measuring the distance between these points, d_100_ was calculated, resulting in a value of 22.8 ± 0.2 Å. This value is in agreement with the calculated one by means of low-angle X-ray diffraction (25 Å, see Table 2).

Nitrogen adsorption–desorption isotherms and BJH of adsorption data of Sn-In-MCM-41, Sn-MCM-41, and In-MCM-41 can be observed in Figures S6 and S7. The isotherm of Sn-In-MCM-41 presents no hysteresis, as have been observed previously in MCM-41 materials with mesopores below 4 nm. Some authors have assigned this isotherm as type II,^56^ according to the IUPAC classification, although it has been also classified as a type I-like isotherm.^57^ The BET specific surface area of Sn-In-MCM-41 is 899 m^2^·g^−1^ with a pore volume of 0.42 cm^3^·g^−1^, which shows that both values are similar to those of Sn-MCM-41 synthesized in the same way.^23^ Nevertheless, these values are slightly lower probably due to the tin and indium atoms inserted into the structure. This could indicate that indium, with a larger size than silicon, slightly decreases the pore size of the MCM-41 structure. On the other hand, textural properties of Sn-MCM-41 and In-MCM-41 are similar to those of Sn-In-MCM-41 (see Figure S6 and Table 2), in agreement with the fact that they were prepared under the same synthetic conditions and have the same hexagonal pore structure. The average pore diameter (Table 2) of Sn-In-MCM-41 is 1.9 nm, similar to Sn-MCM-41 and In-MCM-41.

The BJH analysis (Figure S7) shows similar values to those calculated with 4 V/A equation, which would be according to an MCM-41 structure with homogeneous cylindrical pores. The BJH pore size distribution of Sn-In-MCM-41 is centered at slightly lower values than the other two structures, which is related to its smaller specific surface area and the already mentioned difficulty of introducing indium into the structure, which must in this case compete with Sn. Furthermore, the DFT method was also used and similar pore diameters were obtained.

The pore diameter was also calculated with the method reported by Kruk et al.^54^ using d-spacing and primary volume of mesopores (from t-plot data). A material density of 2.2 g·cm^–3^ was considered for the three catalysts. The results were in agreement with the pore diameters calculated with the abovementioned methods.

**Figure 3:**
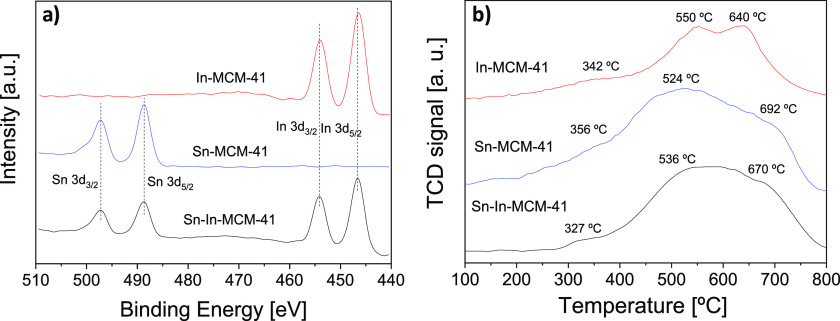
(a) XPS spectra of Sn-MCM-41, In-MCM-41, and Sn-In-MCM-41; (b) TPR profiles of Sn-MCM-41, In-MCM-41, and Sn-In-MCM-41.

XPS was performed for the three catalysts (see Figure 3a). Regarding tin, binding energies for the band 3d_5/2_ of 487.3 and 487.4 eV were registered in Sn-MCM-41 and Sn-In-MCM-41, respectively. These values are in agreement with an oxidation state +4.^50^ In the case of indium, the binding energies of band 3d_5/2_ were 445.5 and 445.4 eV for In-MCM-41 and Sn-In-MCM-41, respectively. This binding energies are related to an oxidation state +3 on zeolites exchanged with indium.^58^ Both spectra of Sn-In-MCM-41 show a lower intensity than those of Sn-MCM-41 and In-MCM-41, in accordance with the lower amount of both metals in this material. The Si/Sn and Si/In atomic ratios calculated by means of XPS data for the three materials are summarized in Table 2. In all cases, the atomic ratios are lower than those obtained by XRF (corresponding to the bulk) and in the synthesis gel, indicating that the proportion of metal atoms in the surface of the catalyst particles is higher than that in the bulk. The same fact was reported by Alarcón et al.^42^ for Sn-MCM-41. This could be due to the ion exchange of OSDA cations with excess cations of tin and indium during the synthesis procedure. XPS shows the elemental composition mainly of the 3 nm nearer to the surface of the particle, using which we can claim that the Lewis acid active sites are very accessible to the reactants. Complete XPS spectra are shown in Figure S8.

Figure 3b shows the TPR profiles of Sn-In-MCM-41, Sn-MCM-41, and In-MCM-41. The three corresponding curves depict the existence of three reduction temperatures. At a temperature of 320–360 °C, the reduction of the most exposed extraframework metal oxides takes place.^59^ In the three cases, the H_2_ consumption is very low and only a soft shoulder can be observed. This is consistent with the fact that most of the metal is in the MCM-41 structure; however, there is a little amount of metal in the particle surface that could contribute to the higher amount of metal determined by XPS (see Table 2). As the intensity of X-ray beam is mainly lost in the surface of the particle, XPS mostly analyzes the first 3 nm closest to the surface of the particles, even though X-rays have a maximum penetration limited to ca. 100 nm.^60^

There are two more reduction peaks at higher temperature due to metal atoms being present in the framework and therefore more difficult to be reduced. The peak at 520–550 °C corresponds to metal atoms near the surface of the framework, while the peak at 640–690 °C corresponds to metal atoms deeper inside the MCM-41 walls, with stronger interactions that are more challenging to be reduced.^42,59^ It can be seen that the reduction peaks of Sn-In-MCM-41 are located at intermediate temperatures between those of Sn-MCM-41 and In-MCM-41, due to the presence of both metals in the structure.

In the TPR profile of In-MCM-41, the peak corresponding to metal atoms within the MCM-41 skeleton appears at lower temperature in comparison to those of Sn-In-MCM-41 and Sn-MCM-41. This is consistent with an easier reduction, in agreement with the fact that the incorporation of indium to the framework is more difficult.

### Catalytic Results

Table 3 lists the results of catalytic tests of sugar conversion to ML carried out with Sn-In-MCM-41 and other catalysts. For the purpose of comparison, catalytic tests were also carried out with tin and indium salts, Sn-MCM-41, In-MCM-41, and mixtures of both catalysts.

**Table 3:** Catalytic Results Obtained for Sugar Conversion with Different Catalysts Using Glucose (160 °C, 20 h, 160 mg of Catalyst and 225 mg of Glucose). Methyl Lactate (ML), Methyl Glycolate (MG), Pyruvaldehyde Dimethyl Acetal (PADA), 1,1,2,2-Tetramethoxypropane (TMP), and Nonidentified Products (N.I.P.). Deviations Shown in the Table Correspond to the Error of 6 Tests for Run 1, 4 Tests for Runs 3, and 2 Tests for Run 4. In the Other Runs, the Deviations Correspond to the Error of Analysis in One Test

run	catalyst	yield (%)					total yield (%)	sugar conv. (%)	TON^d^
		ML	MG	PADA	TMP	n.i.p.			
1	Sn-In-MCM-41	69.4 ± 1.6	1.2 ± 0.3	2.5 ± 0.2	3.1 ± 0.6	4.1 ± 0.3	80.3	>99.7	40.9
2	Sn-MCM-41	46.5 ± 0.5	0.9 ± 0.1	3.9 ± 0.3	2.0 ± 0.1	2.7 ± 0.7	56.0	>99.7	18.3
3	In-MCM-41	26.4 ± 1.6	1.2 ± 0.2	4.4 ± 0.7	0.7 ± 0.0	4.4 ± 0.1	37.1	99.4	19.0
4	Sn-In-MCM-41^a^	73.9 ± 0.8	1.2 ± 0.1	2.2 ± 0.1	2.4 ± 0.3	3.5 ± 0.1	83.2	>99.4	45.9
5	Sn-MCM-41^a^	56.5 ± 2.9	0.3 ± 0.1	1.1 ± 0.5	1.3 ± 0.2	1.9 ± 0.4	61.1	>99.4	23.4
6	SnCl_2_ + InCl_3_	24.1 ± 0.4	1.9 ± 0.1	8.2 ± 0.0	1.0 ± 0.04	3.2 ± 0.1	38.4	98.9	14.2
7	Sn-MCM-41 + In-MCM-41^b^	42.1 ± 1.6	1.6 ± 0.4	4.3 ± 0.4	1.7 ± 0.01	4.2 ± 0.2	53.9	>99.7	21.4
8	Sn-MCM-41 + In-MCM-41^c^	33.8 ± 1.1	1.3 ± 0.3	4.8 ± 0.2	1.5 ± 0.02	4.3 ± 0.3	45.7	>99.7	20.0

a Sugar: sucrose, reaction time: 24 h.

b 80 mg of each one.

c 63.9 mg of Sn-MCM-41 and 78.4 mg of In-MCM-41.

d TON was calculated as mole of ML generated per mol of metal in catalyst at 20 h.

Considering the analyzed reaction products, a reaction mechanism was proposed, and it is given in Scheme S1. The mechanism that leads to the formation of ML includes the isomerization of glucose to fructose, the retro-aldol reaction of fructose to form glyceraldehyde, and dihydroxyacetone and isomerization of these trioses to form ML. Other side reactions of glucose and trioses lead to other products identified in this work.

Except for runs 3 and 4, the concentration of sugar after the reaction was below the detection limit of the determination method, indicating sugar conversions close to 100%. All the ML yields were clearly higher than those of blank experiments carried out previously, 1.1% with glucose, and 1.4% with sucrose^40^ at the same conditions. The difference between the total yield and the conversion of sugar can be attributed to other side reactions, which form humins and other carbonaceous compounds.

Fructose was also analyzed after the reaction, and in all cases, its concentration was under the detection limit of the analytical method. Therefore, retro-aldol reaction of fructose takes place very quickly. Isomerization of glucose to fructose competes with retro-aldol reaction and other side reactions. As a result, the production of ML from fructose usually leads to higher yields than from glucose.^22,28,31^

The experiments carried out with Sn-In-MCM-41 (run 1) reached a mean ML yield of 69.4 ± 1.6%, indicating that this catalyst is very selective for the formation of ML. This value of ML yield is an average of six tests carried out at the same conditions. The low standard deviations given with the ML yields suggest high reliability of the experimental procedure.

The yield to ML obtained with Sn-MCM-41 and In-MCM-41 was 46.5 ± 0.5% (run 2) and 26.4 ± 1.6% (run 3), respectively. The yield obtained with Sn-MCM-41 was similar to that reported previously in the literature (42.7%) with a 3.4 wt % of tin^23^ and a similar but slightly lower turnover number (TON) value (18.3 vs 23.2).

It can be seen in Table 3 that the TON for Sn-In-MCM-41 was more than double those of Sn-MCM-41 and In-MCM-41, and the simultaneous presence of tin and indium improved the catalyst performance. This can be attributed to the fact that tin has high catalytic activity for isomerization of trioses and indium has high catalytic activity for retro-aldol reaction of fructose.^32^ In addition, the presence of indium can reduce side reactions, as was reported in the case of beta zeolite with water as reaction solvent instead of methanol.^37^

In addition to catalytic experiments with glucose, Sn-In-MCM-41 was also used as a catalyst for the conversion of sucrose to ML (run 4), obtaining an ML yield of 73.9 ± 0.8%. This yield is higher than the one obtained with glucose as the substrate (69.4%), in agreement with that reported previously,^40^ and it is due to the fact that the hydrolysis of sucrose decreases the concentration of hexoses in the reaction medium, minimizing side reactions.^40^ The same test was carried out with Sn-MCM-41 (run 5), obtaining an ML yield of 56.5 ± 2.9%. As in the case of glucose, the TON for Sn-In-MCM-41 was approximately double that for Sn-MCM-41, for the same reason as explained above.

With the aim of knowing the effect of the MCM-41 on the activity of tin and indium, an experiment was carried out with tin and indium salts (run 6) using the same amount of each metal as in case of Sn-In-MCM-41. The yield of ML in this experiment was only 24.1%. This value is close to that reported by Zhou et al.:^6^ 26% in the conversion of glucose with SnCl_2_ as a catalyst. This yield is far from that obtained with Sn-In-MCM-41 as the catalyst and with a low TON value of 14.2, thus confirming that the distribution of the metal in the porous material enhances the selectivity of the catalyst. Nemoto et al.^32^ reported that the addition of fluoride salts kept metal cations separated from each other, and as a result, higher yields were obtained. In this case, the mesoporous structure of MCM-41 allows both metals to be independent but very near to catalyze the different steps of the reaction mechanism.

To confirm this hypothesis, another run was carried out with 80 mg of Sn-MCM-41 and 80 mg of In-MCM-41 (run 7) in order to have the same total amount of the catalyst. In this case, the yield to ML was again clearly lower than in the case of Sn-In-MCM-41, 42.1 vs 69.4%. If we consider a linear relationship between the metal mass (either tin or indium) in the catalyst and the yield, the expected yield of this experiment would be 36.5%, which is in the range of the actually obtained yield, 42.1%, as if both separate metals implied summative but not synergistic effects. We also carried out another experiment with 63.9 mg of Sn-MCM-41 and 78.4 mg of In-MCM-41 (run 8) in order to have the same amount of metals as in Sn-In-MCM-41. The obtained ML yield (33.8%) was quite lower than that of run 7 (42.1%) because there was a lower amount of tin, which is more active. In both experiments, TON values were approximately half those for Sn-In-MCM-41. In addition, Nemoto et al.^32^ reached maximum yields of ML of 49% with glucose as a substrate and of 55% with sucrose, using indium and tin chlorides at 160 °C. Both values are lower than those achieved in this work, suggesting that an even distribution of tin and indium throughout the mesoporous material favors a synergistic effect of both metals better than that of the homogeneous catalyst.

Table 4 summarizes the best results found in the literature for the conversion of sugars to ML with a heterogeneous catalyst at similar reaction conditions. Since Holm et al. reported results using zeolite beta as catalysts,^22^ several improvements have been made, such as the introduction of alkaline ions in the zeolite synthesis.^24^ Zhang et al.^26^ reported yields to ML of 52.5 and 72.1% with glucose and sucrose as substrates, respectively, using a hierarchical Sn-beta zeolite as a catalyst. Recently, Tang et al.^29^ used another Sn-beta catalyst and reached a 52% yield from glucose and a 69% yield from sucrose. Per our knowledge, these are the highest yields reached for this reaction. It is worth mentioning that Tolborg et al.^24^ reported in the conversion of sucrose an ML with a yield of 75%, higher than those indicated in Table 4, but obtained by adding K_2_CO_3_ (0.065 mM) to the reaction medium.

**Table 4:** Literature Review of Methyl Lactate Yield from Glucose and Sucrose at 160 °C Using Heterogeneous Catalysts

catalyst	ML yield (%)		ref
	with glucose	with sucrose	
Sn-beta	43.0	64.0	(22)
Sn-MWW	49.0	55.0	(13)
Rb-Sn-beta		67.0	(24)
hierarchical Sn-beta	52.5	72.1	(26)
Sn-beta-WO_3_	52.0	60.0	(27)
Sn-beta-H	52.0	69.0	(29)
In-Sn-Beta zeolite	53.0^a^		(37)
Zn-Sn-Beta zeolite	57.2^b^		(38)
Sn-MCM-41	46.5	56.5	this work
Sn-In-MCM-41	69.4	73.9	this work

a Yield to lactic acid.

b Temperature: 150 °C.

We can claim that the results obtained with Sn-In-MCM-41 overcome the previous ones either for glucose or sucrose as a substrate. Besides, it is worth mentioning that total yields (including the main product ML but also MG, PADA, TMP, and other nonidentified products, see Table 3) are 80.3% (69.4% to ML) and 83.2% (73.9% to ML) from glucose and sucrose, respectively. Values that suggest that the exploitation of the two substrates is remarkable when using In-Sn-MCM-41.

### Reusability of Sn-In-MCM-41

**Figure 4:**
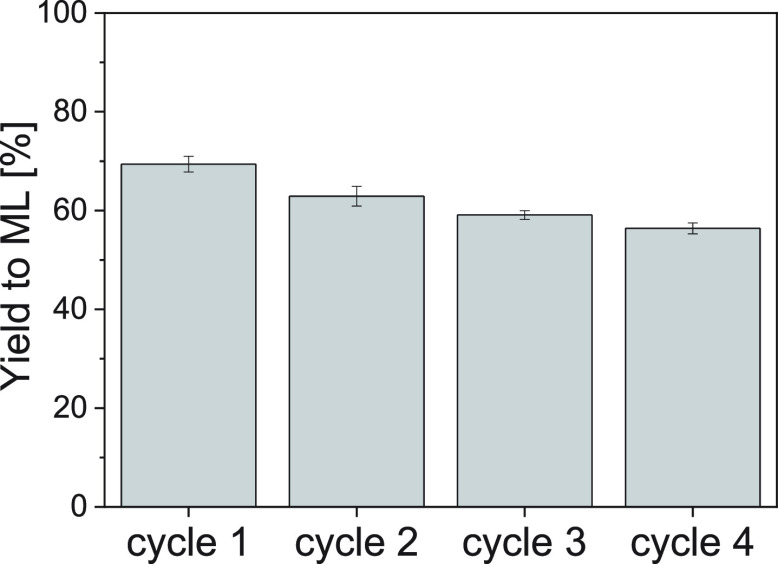
Yield of methyl lactate in the conversion of glucose with Sn-In-MCM-41 up to 4 catalytic cycles (160 °C for 20 h, 160 mg of the catalyst, and 225 mg of glucose). Error bars correspond to deviations in six runs for cycle 1, four runs for cycle 2, two runs for cycle 3, and deviation in the mass spectrometry chromatographic analysis in one run for cycle 4.

To evaluate the reusability of the catalyst, Sn-In-MCM-41 was tested in four consecutive catalytic cycles (results of all the cycles are summarized in Table S1). In all cases, the conversion of sugar was close to 100%, indicating that the activity was maintained. Nevertheless, the selectivity decreased appreciably, and consequently, the yield to ML decreased from 69.4 to 56.4% at the latest cycle (see Figure 4), while the yields to MG, PADA, and TMP were kept constant or even slightly increased. Thus, a similar variation to ML yield was observed for the total yield. Even though such descent, the values of ML and total yield are still very high and the decrease is softened with the cycles.

**Figure 5:**
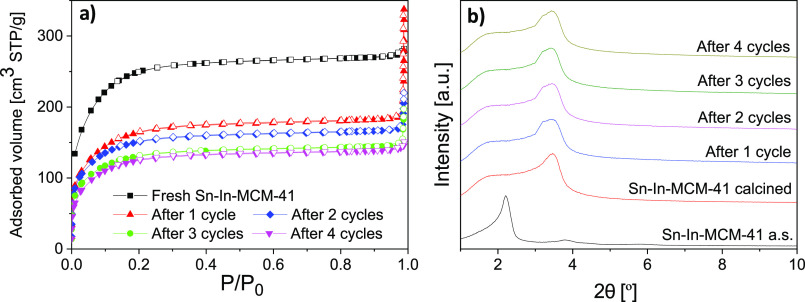
(a) Nitrogen adsorption–desorption isotherms of fresh Sn-In-MCM-41 and reutilized up to four catalytic cycles; (b) XRD patterns of Sn-In-MCM-41 as synthesized (a.s.), calcined, and reutilized up to four catalytic cycles.

With the aim of studying the stability, the catalyst was characterized after its use in glucose conversion. Figure 5a shows the nitrogen adsorption–desorption isotherms of Sn-In-MCM-41 fresh and after the four catalytic cycles. After the first use of the catalyst, the adsorbed volume suffered a high descent, which coincides with a decrease in the BET specific surface area and pore volume (see Table 5). This reduction can be attributed to the fact that some products are adsorbed in the porous structure of the catalyst, and they are not removed during the degasification carried out prior to nitrogen adsorption measurement. This result is in agreement with the fact that the higher decrease of ML yield took place after the first cycle, from 69.4 to 62.9% (a 6.5% of yield loss). After the following cycles, an increase in adsorbed products could be detected by the values of the BET specific surface area and pore volume, but the differences between these values are not as high as for the first cycle. Furthermore, the reduction of ML yield was also lower, 3.8% between second and third cycle, and 2.7% between third and fourth.

**Table 5:** Properties of Fresh Sn-In-MCM-41 and after Each Catalytic Cycle

	fresh	cycle 1	cycle 2	cycle 3	cycle 4
BET surface area [m^2^·g^−1^]	899 ± 15	595 ± 8	536 ± 9	465 ± 7	448 ± 7
pore volume^a^ [cm^3^·g^−1^]	0.42	0.29	0.26	0.23	0.22
pore diameter^b^ [nm]	1.90	1.93	1.96	1.96	1.95
Sn XRF [wt %]	1.88 ± 0.04	1.74 ± 0.04	1.72 ± 0.05	1.67 ± 0.03	1.64 ± 0.04
In XRF [wt %]	1.22 ± 0.02	1.13 ± 0.02	1.10 ± 0.01	1.08 ± 0.01	1.06 ± 0.02
Si/Sn XRF	102	109	110	114	116
Si/In XRF	152	163	167	170	174
Si/Sn XPS	52				67
Si/In XPS	23				46

a At *P*/*P*_0_ = 0.97.

b 4 V/A by BET.

Low-angle X-ray diffractograms of Sn-In-MCM-41 fresh and after the catalytic cycles are depicted in Figure 5b. It can be observed that the diffraction peak corresponding to (100) planes widened after being used for the first time. Therefore, we cannot discard that the results of the nitrogen adsorption could be also due to a structure deterioration. No notable changes are observed in the other diffractograms of the catalyst after the remaining cycles.

During the reaction cycles, a slight loss of metal (Sn and In) present in the catalyst takes place (see Table 5). This can be attributed to the presence of the low amount of oxide particles. The TONs of these catalytic cycles are available in Table S1. It is maintained around 38 up to the fourth cycle (with a value of 37.6). Then, up to this cycle, the decrease in ML yield can be attributed with an important role to the loss of metals. Therefore, it can be concluded that the structural order of Sn-In-MCM-41 is practically maintained even after four catalytic cycles.

XPS of Sn-In-MCM-41 is shown in Figure S9. Binding energy of 3d_5/2_ of tin for Sn-In-MCM-41 after four cycles is 487.3 eV, similar to that of the fresh catalyst. The intensity corresponding to the oxidation state +4 was lower due to the loss of tin during reaction; in fact, the Si/Sn ratio changed from 52 (fresh) to 67 (after four cycles). For indium, the binding energy of band 3d_5/2_ is 444.8 eV, corresponding to an oxidation state +3. No reduction of metal cations was observed in the presence of glucose that is a reducing sugar. The Si/Sn and Si/In ratios determined by XPS increased after the fourth cycle. Nevertheless, the ratios were lower than those determined by XRF, indicating a higher amount of metals in the surface of Sn-In-MCM-41 particles, as discussed above.

In the literature, bimetallic catalysts have also been recycled for the transformation of glucose with a slight decrease in the yield;^38,39^ for example, using hierarchical zeolite Zn-Fe-beta, the ML at 220 °C yield changed from 67 to ca. 60% after five cycles. For example, using hierarchical zeolite Zn-Fe-beta, the ML at 220 °C yield changed from 67 to ca. 60% after five cycles.^38^ Such a decrease was related to changes in the porous structure and surface chemistry. It should be noted that in these studies with zeolite beta, the catalyst was calcined between cycles at 550 °C to remove carbonaceous deposits. In our case, we have opted for a lower temperature process with a treatment between cycles at 70 °C.

### LCA Results

In order to evaluate the environmental impact of the route for the production of ML proposed in this work, LCA was carried out. The functional unit of this study is the production of 1 kg of ML, analyzing the EII of biochemical route and chemical route with Sn-MCM-41 and Sn-In-MCM-41 as catalysts. Data for the biochemical route extracted from bibliography are for the production of 1 kg of LA, which is obtained after ML production by esterification and hydrolysis. Methanol for esterification is recovered and reused in the process. In consequence, the low amount used (0.02 kg, Table 1) does not have a relevant influence on the LCA results. However, the energy data for the biochemical route may be overestimated in Table 1. For this reason, a Monte Carlo analysis involving 100 simulations has been performed to estimate uncertainty variations of ±10% in the thermal energy input for the biochemical route. The objective of this sensitivity analysis was to evaluate if LCA results were significantly affected by uncertainties on thermal energy input data. Results of this analysis show coefficients of variation for the EIIs below 5%. This allows us to conclude that the biochemical route data can be used with enough confidence. Because in both chemical routes the ML yield varies during the catalytic cycles, we considered that the catalyst was reused four times with an averaged glucose yield to ML of 40 and 62% for Sn-MCM-41 and Sn-In-MCM-41 catalysts, respectively.

**Figure 6:**
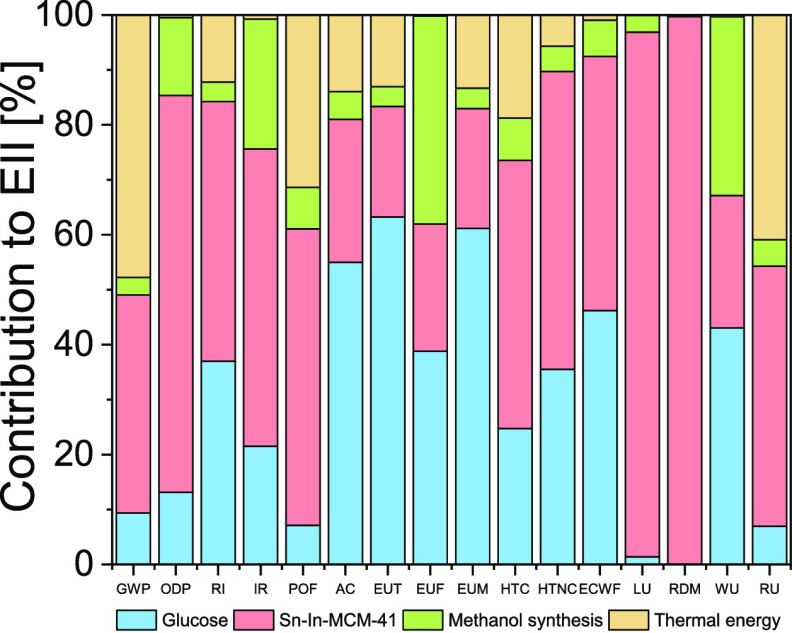
Contribution of the different processes to the environmental impact indicators values for the chemical route (Sn-In-MCM-41). GWP: global warming potential [kg CO_2_ eq]; ODP: ozone layer depletion [kg CFC-11 eq]; RI: respiratory inorganics [disease incidences]; IR: ionizing radiation–human health [kBq U-235 eq]; POF: photochemical ozone formation–human health [kg NMVOC eq]; AC: acidification terrestrial and freshwater [Mole H^+^ eq]; EUT: eutrophication terrestrial [Mole N eq]; EUF: eutrophication freshwater [kg P eq]; EUM: eutrophication marine [kg N eq]; HTC: human toxicity potential, cancer effects [CTUh]; HTNC: human toxicity potential, non-cancer effects [CTUh]; ECFW: ecotoxicity freshwater [CTUe]; LU: land use [Pt]; RDM: resource use, mineral, and metals [kg Sb eq]; WU: water use [m^3^ world eq]; RU: resource use and energy carriers [MJ].

Figure 6 and Table S6 show values of the indicators for the Sn-In-MCM-41 chemical route, detailing the contributions to the total value of the different processes. It can be seen in Figure 6 that the main responsible for Sn-In-MCM-41 chemical route EIIs values are catalyst synthesis for 10 of 16 EIIs, glucose for 4 of 16, methanol production for 1 of 16, and thermal energy for 1 of 16. The main reason of these impacts is the high amount of catalyst and reactants used (Table 1). Tables S4 and S5 and Figures S11 and S12 show results for biochemical and Sn-MCM-41 routes, respectively.

From these data, we can say that, either for the biochemical or chemical route, raw glucose production and the thermal energy are mainly responsible for their EII values. Nevertheless, both impacts are more important in the chemical route because of the higher amounts of glucose and methanol and higher temperature required (see Table 1). Regarding exclusively the chemical routes (either with Sn-MCM-41 or Sn-In-MCM-41 as catalysts), catalyst synthesis and methanol synthesis and recuperation have also important contribution to their EII values.

**Figure 7:**
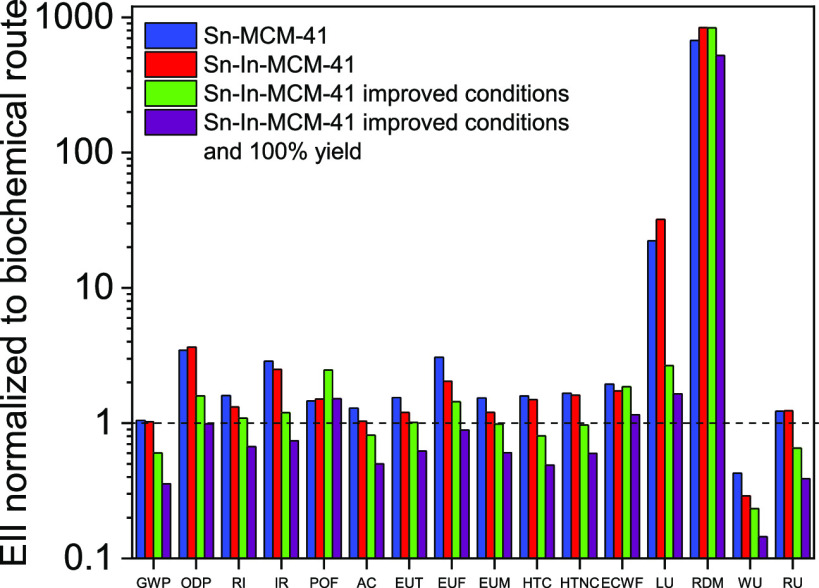
Environmental impact indicator variations of the different routes normalized to the biochemical route. Definition of EIIs in Figure 6 caption.

The variation of the EIIs for the chemical routes relative to the biochemical route are represented in Figure 7. The use of Sn-In-MCM-41 as a catalyst reduces the values of the 16 EII studied compared to Sn-MCM-41 due to the increase in ML reached with this new catalyst, which implies a lower consumption of reactants. In spite of this improvement, the most industrially used biochemical route for the production of ML has lower impacts than the chemical routes.

Several improvements have been proposed to reduce the most important impacts of the Sn-In MCM-41 chemical route. Because catalyst synthesis is mainly responsible for 10 of 16 EIIs (Figure 6), improvements have to be mainly focused on the reduction of these impacts. An obvious possibility is to improve the catalytic productivity (g of ML per g of catalyst and hour). Another possible improvement would be to reduce the catalyst amount used during the reaction. However, from authors’ previous studies for ML synthesis from sugars with ZIF-8^40^ and with UZAR-S4^41^ as catalysts, catalyst yield reduction was observed with the decrease of the catalyst amount. For this reason, the amount of 160 mg has been kept here as the lowest, below which the catalyst activity is reduced. Another obvious improvement would be to increase the number of cycles so that the catalyst could be reused from 4 to 12 times. This study has been done for the Sn-MCM-41 catalyst. Because of the decrease of catalyst activity toward ML from 4 to 12 times recycling not obtained experimentally, a linear decrease was calculated for the purpose of LCA estimations. In these conditions, an average catalytic activity toward ML for the 12 times recycled Sn-MCM-41 catalyst of 31.23% was obtained. With this conversion, the amounts of glucose and methanol needed are 2.76 and 4.91 kg, respectively, and although the catalyst needed is only 6.66 mg, the environmental impact reduction produced by the less catalyst amount used is comparatively lower than the environmental impact increment due to the higher glucose and methanol amounts used. Hence, it can be concluded that an increase in the number of times the catalyst is reused from 4 to 12 times is not beneficial in terms of environmental impacts. However, it is worth noting that the influence on the reaction yield and on EIIs of the reduction of the Sn-In-MCM-41 catalyst amount and of the increase on the number of times it is reused has to be experimentally studied and will be the object of a future work. On the other hand, the high impact of the catalyst synthesis is mainly due to the synthesis of TEOS (data not shown). This effect has also been observed in LCA applied to zeolite synthesis.^61^ Because of this, to reduce catalyst synthesis impacts, TEOS was substituted in the LCA calculation by Na_2_SiO_3_; in fact, this option has been demonstrated as feasible by several authors.^62,63^ Other important impact is the thermal energy, which is mainly used to heat the methanol of the reaction medium to a temperature of 160 °C (see experimental section). Therefore, to decrease the thermal energy impact, the amount of methanol used during the ML synthesis was reduced in the LCA estimation by half. With this proposal, the impact corresponding to the methanol synthesis and recuperation impact would be also reduced.

With these two modifications (TEOS substitution to Na_2_SiO_3_ in catalyst synthesis and methanol reduction in ML synthesis; see inventory data on Table 1), the EII values were notably diminished (Table S3, Figure 7, green columns), but most of them are still above those corresponding to the biochemical route impacts. As a final approach to decrease even more the EII values of the chemical route, the Sn-In-MCM-41 catalyst yield to ML was ideally increased to 100%. This would reduce significantly the amounts of glucose and catalyst used, which are the most important contributors to EIIs (see Figure 6 and inventory data on Table 1). As can be seen in Table S3 and Figure 7 (purple columns), under these circumstances, all EIIs of the chemical process except one (RDM) are lower than those of the biochemical route.

In summary, this LCA study opens a wide number of possibilities to reduce the impacts of ML synthesis and, as it has been proven, methanol reduction to the half, TEOS substitution to Na_2_SiO_3_, and 100% catalyst yield are effective improvements. These measures, as well as others that can also reduce EII values like the catalyst amount and synthesis time reduction, have to be tested in a laboratory. It is worth mentioning that all these improvements would require further efforts in the catalyst synthesis and in the process optimization, which are beyond the scope of this work.

## Conclusions

Sn-In-MCM-41 was successfully synthesized for the first time with Si/Sn and Si/In atomic ratios of 101 and 151 (Si/metal = 61), respectively, as determined by XRF. To study the properties of this new material, Sn-MCM-41 and In-MCM-41 were also synthesized. The XRD and TEM characterizations of Sn-In-MCM-41 showed that the material has an MCM-41 mesoporous structure with a homogeneous distribution of both metals. By means of XPS, it was observed that the indium and tin atoms in the material have oxidation states of +3 and +4, respectively. Most of the metallic charge is part of the MCM-41 structure, although XPS revealed a higher concentration of metals on the surface and/or the mesoporosity of the solid. Sn-In-MCM-41 possesses Lewis and Brønsted acid sites due to the presence of metal cations and silanol groups, respectively, and this fact was confirmed by titration and by the analysis of physisorbed pyridine coupled with FTIR spectroscopy.

The synthesized material was applied as a catalyst to the conversion of sugars with high activity and selectivity to ML. Yields to ML of 69.4% in the transformation of glucose and of 73.9% in the transformation of sucrose were reached, suggesting a synergistic effect when both metals tin and indium were combined in the same material. In fact, neither the simple mix of Sn-MCM-41 and In-MCM-41 nor that of the corresponding chlorides were able to surpass the performance of Sn-In-MCM-41. These are the highest ML yields reported in the literature to date for heterogeneous catalysts at similar reaction conditions. Regarding its stability, Sn-In-MCM-41 was reused in four catalytic cycles, with a slight reduction of ML yield, mainly during the first cycle. The characterization of the catalyst before and after the reaction cycles suggests that the loss of activity toward ML was mainly due to the reduction of the textural properties, attributed to the nonreversible adsorption of reaction products in the catalyst and also to the slight metal leaching. Finally, regarding LCA results, it can be concluded that the use of Sn-In-MCM-41 reduces the environmental impacts compared to Sn-MCM-41. In addition, by means of LCA, new possibilities in terms of research of catalyst synthesis and process optimization have been opened to make the impacts of the chemical route comparable to those of the more favourable biochemical route.

## Supporting Information Available

TGA curves of Sn-In-MCM-41: as synthesized, calcined, and after four catalytic cycles; XRD patterns of as-synthesized and calcined Sn-In-MCM-41; XRD patterns of as-synthesized and calcined In-MCM-41; FTIR spectra of calcined Sn-In-MCM-41, Sn-MCM-41, and In-MCM-41; FTIR spectra of Sn-In-MCM-41, Sn-MCM-41, and In-MCM-41 before and after pyridine adsorption and heating at 353 K; nitrogen adsorption–desorption isotherms of Sn-In-MCM-41, Sn-MCM-41, and In-MCM-41; BJH adsorption of Sn-In-MCM-41, Sn-MCM-41, and In-MCM-41; complete XPS spectra of Sn-In-MCM-41 fresh and after four catalytic cycles, Sn-MCM-41, and In-MCM-41; XPS spectra of Sn-MCM-41 fresh and after four catalytic cycles; catalytic results obtained for sugar conversion with Sn-In-MCM-41 in catalytic cycles (160 °C for 20 h, 160 mg of catalyst, and 225 mg of glucose); reaction mechanism; inputs for the LCA calculations on GaBi Software; flowchart for the production of 1 kg of glucose (Ecoinvent); environmental impact indicators (EIIs), units, and recommendation level; environmental impact indicator values; and contribution of the different processes to the EII values for the biochemical and chemical routes (PDF)
