# Supplementary material for: Mesoporous Sn-In-MCM-41 Catalysts for the Selective Sugar Conversion to Methyl Lactate and Comparative Life Cycle Assessment with the Biochemical Process
Source: ACS Sustain Chem Eng. 2022 Feb 21;10(9):2868–80. doi: 10.1021/acssuschemeng.1c04655 (PMC8906110; doi:10.1021/acssuschemeng.1c04655)
Supplement: Supplementary file 1 — sc1c04655_si_001.pdf [file sc1c04655_si_001.pdf]

Mesoporous Sn-In-MCM-41 catalysts for the selective sugar conversion to methyl lactate and comparative life cycle assessment with the biochemical process

Óscar de la Iglesia [1,2] *, Miryan Sarango [2,3], Mikel Munárriz [4], Magdalena Malankowska [2,3], Alberto Navajas [4,5], Luis M. Gandía [4,5], Joaquín Coronas [2,3], Carlos Téllez [2,3] #.

[1] Centro Universitario de la Defensa Zaragoza, Academia General Militar, 50090 Zaragoza, Spain.

[2] Instituto de Nanociencia y Materiales de Aragón (INMA), CSIC-Universidad de Zaragoza, 50018 Zaragoza, Spain.

[3] Department of Chemical and Environmental Engineering, Universidad de Zaragoza, 50018 Zaragoza, Spain.

[4] Department of Science, Universidad Pública de Navarra, Campus de Arrosadia, 31006 Pamplona, Spain.

[5] Institute for Advanced Materials and Mathematics (InaMat2), Universidad Pública de Navarra, Edificio Jerónimo de Ayanz, Campus de Arrosadia, 31006 Pamplona, Spain.

* E-mail: oiglesia@unizar.es.

# E-mail: ctellez@unizar.es.

Supporting Information contents: 16 pages, 12 figures, 6 tables and 1 scheme.

SUPPORTING INFORMATION

List of figures, tables and schemes

Figure S1 ....S2
Figure S2 ....S2
Figure S3 ....S3
Figure S4 ....S3
Figure S5 ....S4
Figure S6 ....S4
Figure S7 ....S5
Figure S8 ....S5
Figure S9 ....S6
Table S1 ....S6
Scheme S1....................S7
Figure S10 ....S8
Table S2 ....S10
Table S3 ....S11
Table S4 ....S12
Table S5 ....S13
Table S6 ....S14
Figure S11 ....S15
Figure S12 ....S16

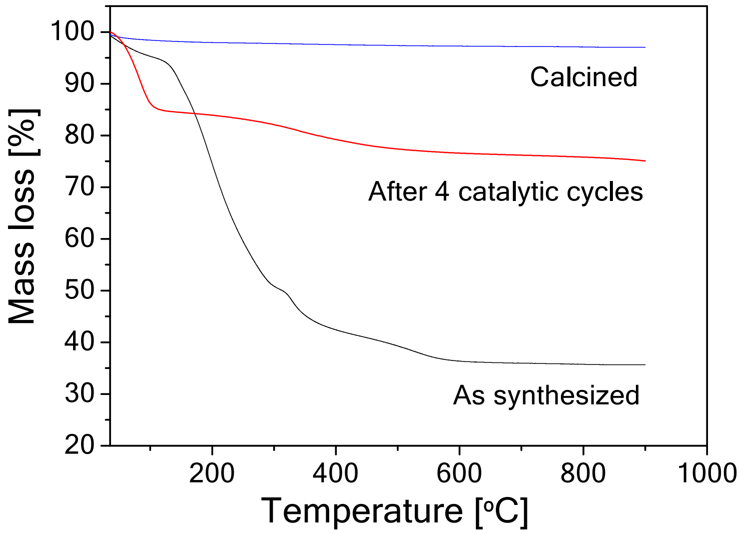

Figure S1. TGA curves of Sn-In-MCM-41: as synthesized; calcined and after 4 catalytic cycles.

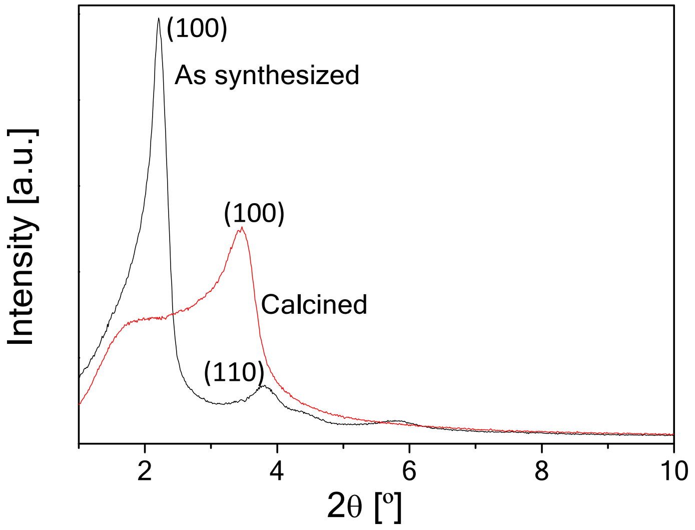

Figure S2. XRD patterns of as synthesized and calcined Sn-In-MCM-41.

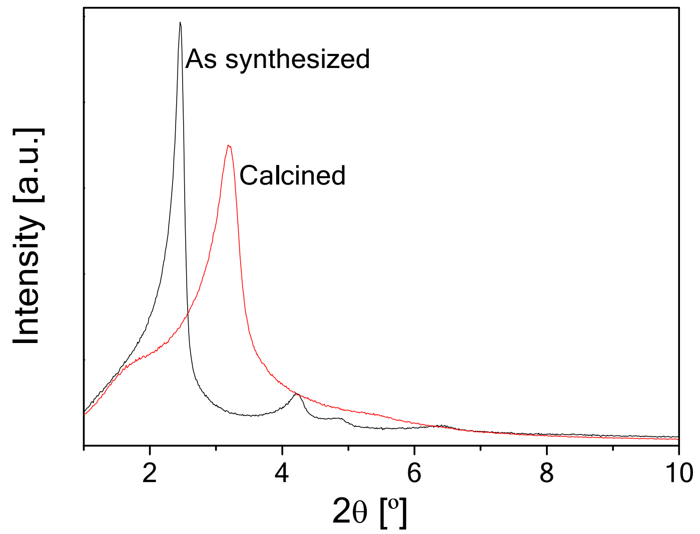

Figure S3. XRD patterns of as synthesized and calcined In-MCM-41.

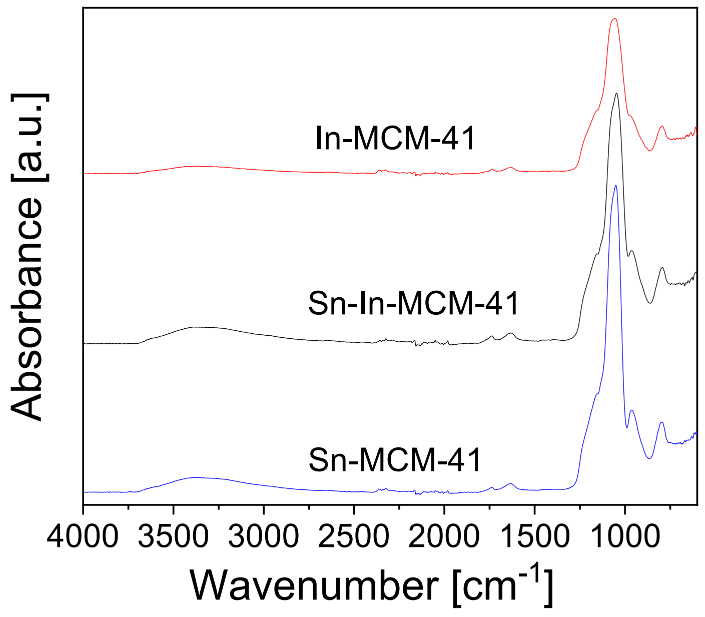

Figure S4. FTIR spectra of calcined Sn-In-MCM-41, Sn-MCM-41 and In-MCM-41.

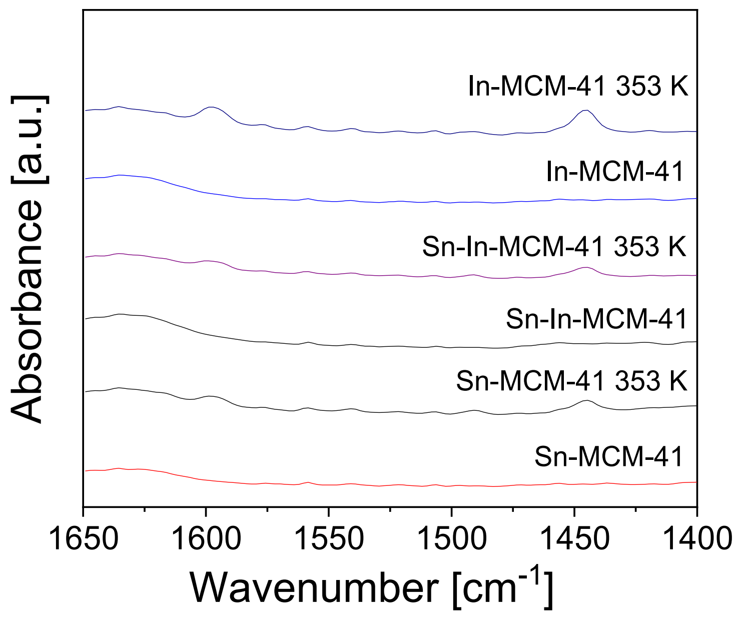

Figure S5. FTIR spectra of Sn-In-MCM-41, Sn-MCM-41 and In-MCM-41 before and after pyridine adsorption and heating at 353 K.

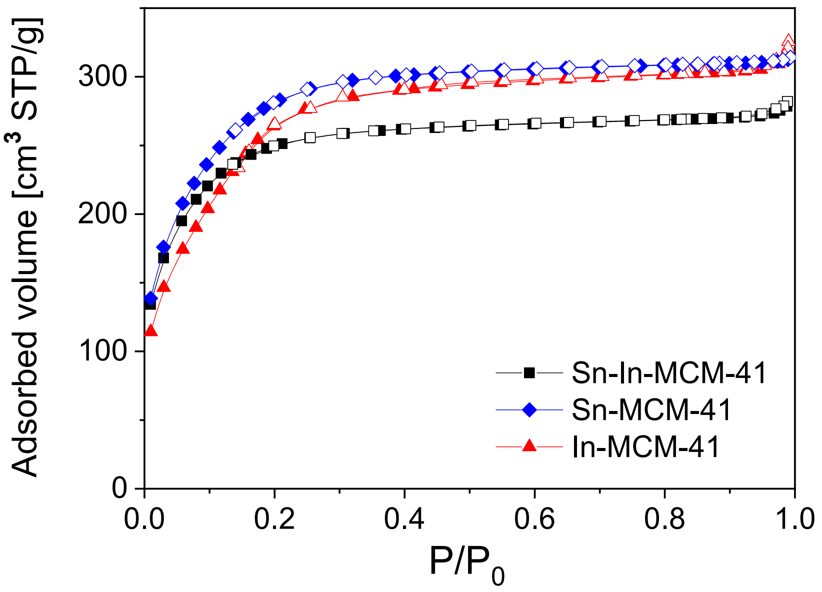

Figure S6. Nitrogen adsorption-desorption isotherms of Sn-In-MCM-41, Sn-MCM-41 and In-MCM-41.

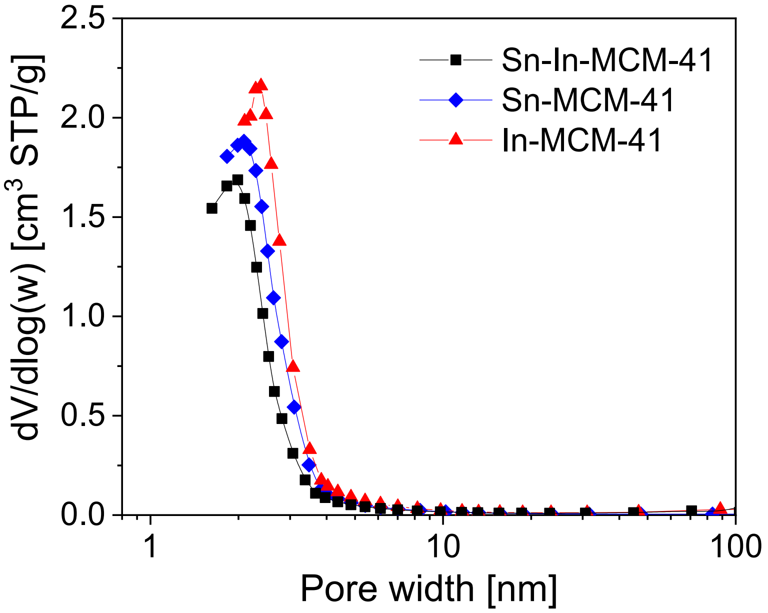

Figure S7. BJH adsorption of Sn-In-MCM-41, Sn-MCM-41 and In-MCM-41.

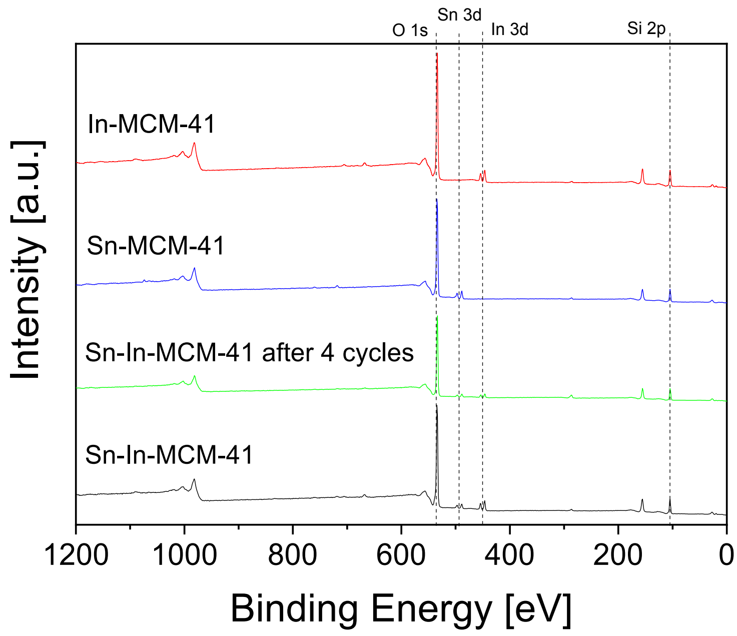

Figure S8. Complete XPS spectra of Sn-In-MCM-41 fresh and after 4 catalytic cycles, Sn-MCM-41 and In-MCM-41.

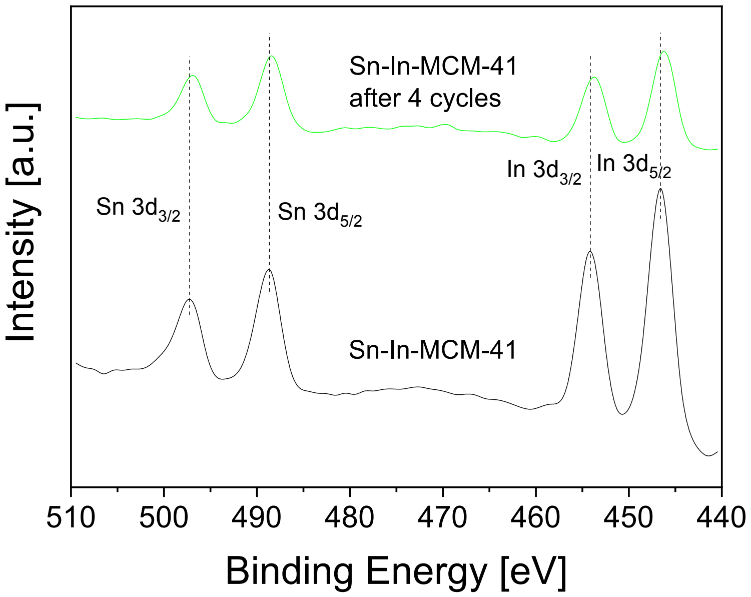

Figure S9. XPS spectra of Sn-MCM-41 fresh and after 4 catalytic cycles: a) Sn 3d and b) In 3d.

Table S1. Catalytic results obtained for sugar conversion with Sn-In-MCM-41 in catalytic cycles using glucose (160 °C for 20 h, 160 mg of catalyst and 225 mg of glucose). Methyl lactate (ML), methyl glycolate (MG), pyruvaldehyde dimethyl acetal (PADA), 1,1,2,2-tetramethoxypropane (TMP) and non-identified products (n.i.p.). Deviations correspond to the error of 6 test for cycle 1, 4 tests for cycle 2, 2 tests for cycle 3 and the error in the analysis in one test for cycle 4.

Catalytic Cycle	Yield (%)					Total yield (%)	Sugar conv. (%)	TON[a]
	ML	MG	PADA	TMP	n.i.p.			
1	69.4±1.6	1.2±0.3	2.5±0.2	3.1±0.6	4.1±0.3	80.3	>99.7	41.0
2	62.9±2.0	1.6±0.2	1.6±0.1	2.3±0.2	3.6±0.3	72.0	>99.7	40.0
3	59.1±0.9	1.5±0.07	1.9±0.1	1.9±0.1	3.4±0.3	67.8	>99.7	38.4
4	56.4±1.1	1.5±0.1	2.3±0.4	1.6±0.1	3.2±0.2	65.0	>99.7	37.6

[a] TON was calculated as mole of ML generated per mol of metal in catalyst at 20 hours.

Scheme S1. Reaction mechanism.

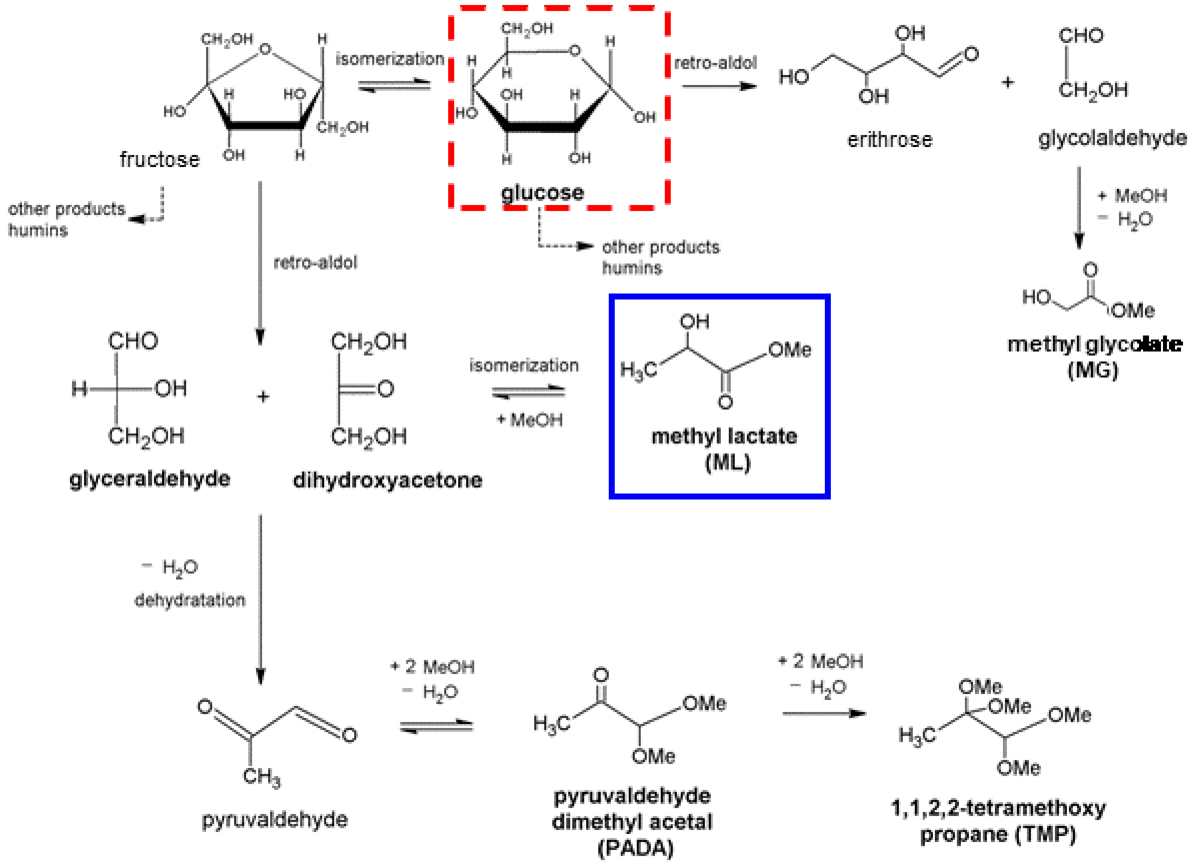

Inputs for the LCA calculations on GaBi Software

Biochemical route

In this section, inputs whose impacts are not included in GaBi database will be explained. Methanol has been simulated using data from Chen et al.[1] As nutrients it has been supposed that ammonia is used. Regarding the bacteria, the protein production of unicellular beings from hexadecane has been considered.[2] Dry bacteria are the proteins generated during cellular respiration and, therefore, to produce 10.6 moles of dry biomass from bacteria, 1 mole of hexadecane, 12.5 of oxygen and 2.13 of ammonia have been used (1).

$$C_{16}H_{34} + 12.5\ O_2 + 2.13\ NH_3 \rightarrow 10.6\ CH_{1.66}O_{0.27}N_{0.20} + 5.37\ CO_2 + 11.4\ H_2O \quad (1)$$

For the modeling of glucose production, data have been taken from the Ecoinvent database. The data used are shown in Figure S10.

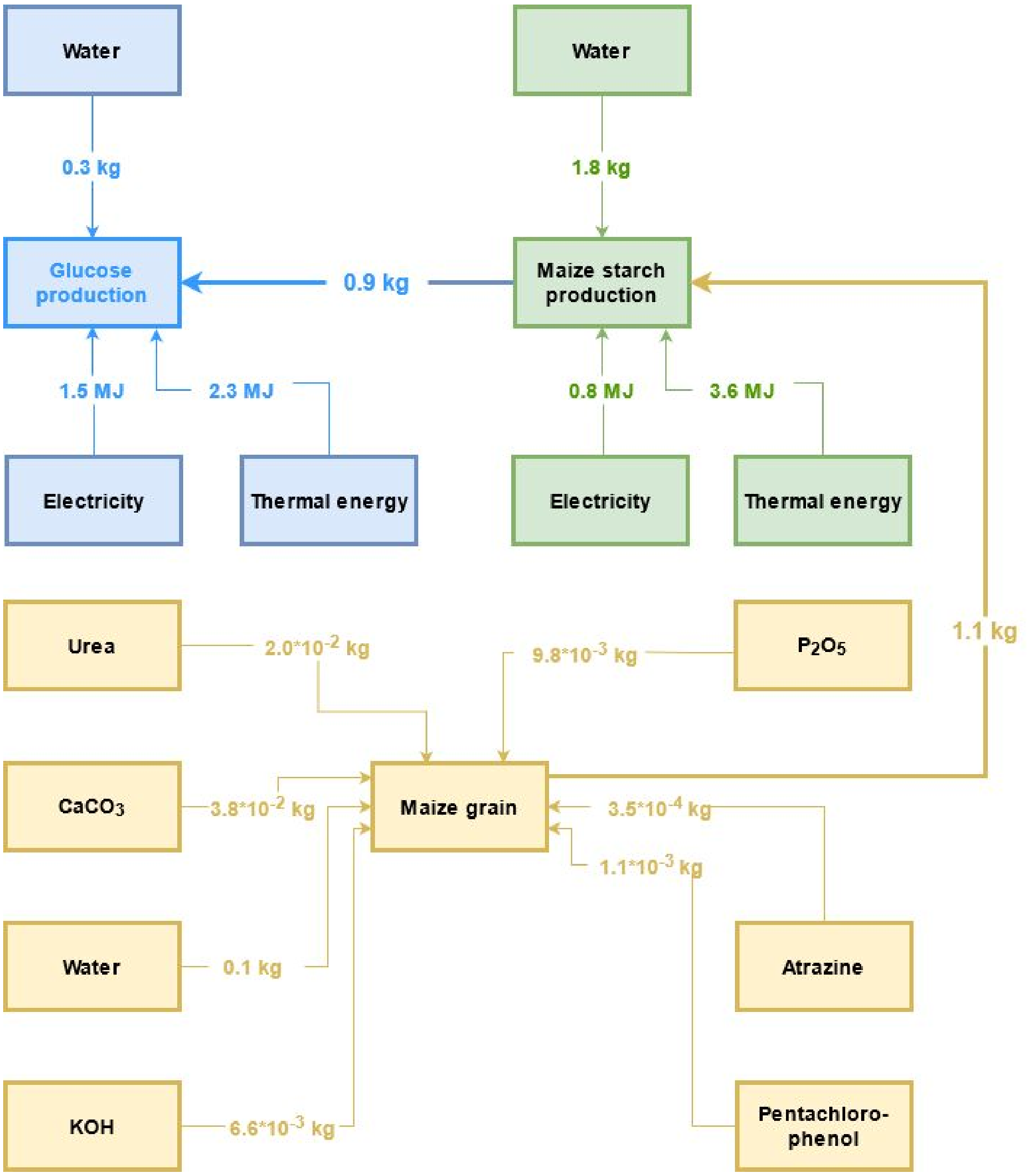

Figure S10. Flowchart for the production of 1 kg of glucose (Ecoinvent).

Urea has been considered to be produced by the reaction of 2 moles of ammonia with 1 mole of CO_2 (2, 3).

$$2\,NH_3 + CO_2 \leftrightarrow H_2NCOONH_4 \qquad (2)$$
$$H_2NCOONH_4 \leftrightarrow (NH_2)_2CO + H_2O \qquad (3)$$

The KOH production process does not exist in the GaBi databases, but it does exist in Ecoinvent. As fertilizers, phosphorus oxide (V) has been mainly used, which does not appear in GaBi database, but it appears in the Ecoinvent database. Atrazine is a herbicide that does not appear in the GaBi database and its production has been modeled by the reaction of hydrochloric cyanide with one equivalent of ethylamine, followed by treatment with one equivalent of isopropylamine.[3] Ethylamine and isopropylamine have been assimilated as dimethyl amine, which has been modeled taking into account that to obtain one mole, 1 mole

of ammonia and 2 moles of methanol are needed. For the synthesis of hydrochloric cyanide, it has been considered that it is synthesized in two steps, the first is the production of cyanogen chloride and then its trimerization (4, 5).[4]

$$HCN + Cl_2 \rightarrow ClCN + HCl \quad (4)$$

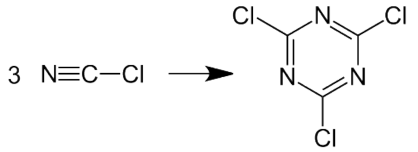

(5)

Therefore, to produce one mole of hydrochloric cyanide, it has been considered that 3 moles of Cl_2 and 3 moles of HCN (prussic acid) are needed, which do appear in the GaBi database. Finally, pentachlorophenol is a pesticide that is synthesized with phenol and chlorine (6), and phenol production is included in the GaBi database.

$$C_6H_5OH + \frac{5}{2}Cl_2 \rightarrow C_6HCl_5O + \frac{5}{2}H_2 \quad (6)$$

Chemical route

TEOS is simulated by the reaction between tetrachlorosilane and ethanol[4] (7).

$$SiCl_4 + 4\ C_2H_5O_2H \rightarrow Si(OC_2H_5)_4 + 4\ HCl \quad (7)$$

Ethanol synthesis process is in-built in GaBi database and is described by the hydration of ethylene in a gas phase reactor with nitric acid as catalyst. $SiCl_4$ industrial synthesis is produced by reaction of Si and chlorine.[5] Both appear in GaBi database.

Cetrimonium bromide (CTABr), $CH_3(CH_2)_{15}N(Br)(CH_3)_3$, has been considered to be synthesized by the addition of 1 mole of hexadecane, 1 mole of triethylamine and 1 mole of bromine. Hexadecane appears in GaBi databse, triethyl amine has been assimilated as dimethylamine, and bromine has been simulated using Ecoinvent database.

$SnCl_2$ has been simulated using Sn and HCl. HCl is in GaBi database and tin production has been simulated using Ecoinvent database. Finally $InCl_3$ has been simalted by the same way. Indium has been considered to be a subproduct of the zinc industry.[6]

It should be pointed out that 95% methanol in the chemical route has been considered to be reused in the four sucesive reutilizations of the catalyst by distillation.

Table S2. Environmental impact indicators (EIIs), units and recommendation level.[7]

Environmental Indicator	Units	Classification
Global Warming Potential (GWP)	kg CO_2 Eq.	I
Ozone Depletion Potential (ODP)	kg CFC-11 Eq.	I
Respiratory inorganics (IR)	kg PM2,5 Eq.	I
Ionizing radiation, human health effect model (IR)	kg U-235 Eq.	II
Photochemical ozone formation (POF)	kg NMVOC Eq.	II
Acidification Potential (AC), accumulated exceedance.	Mole of H^+ Eq.	II
Terrestrial Eutrophication (EUT)	Mole f N Eq.	II
Freshwater Eutrophication (EUF)	kg of P Eq.	II
Aquatic marine Eutrophication (EUM)	kg of N Eq.	II
Human Toxicity Potential, Cancer effects (HTC)	Comparative Toxic Unit for Human Health (CTUh)	III
Human Toxicity Potential, Non-Cancer Effects (HTNC)	Comparative Toxic Unit for Human Health (CTUh)	III
Ecotoxicity freshwater (ECFW)	Comparative Toxic Unit for ecosystems (CTUe)	III
Land Use (LU)	Soil Quality Index (Pt)	III
Resource use, mineral and metals (RDM)	kg Sb Eq.	III
Water Use (WU)	m^3 Eq.	III
Resource use, energy carriers (RU)	MJ Eq.	III

Table S3. Environmental impact indicators (EIIs) values for the biochemical and chemical routes, Sn-In-MCM-41 with Na_2SiO_3 as silicium source and ½ MeOH, and Sn-In-MCM-41 with Na_2SiO_3 as silicium source, ½ MeOH, and 100% yield from glucose to ML.

EI	Ud.	Biochemical route	Sn-MCM-41	Sn-In-MCM-41	Sn-In-MCM-41 (Na2SiO3+1/2 MeOH)	Sn-In-MCM-41 (Na2SiO3+1/2 MeOH) 100 % Yield
GWP	kg CO2 eq.	12.4	13.0	12.6	7.5	4.4
ODP	kg CFC-11 eq.	$1.21\cdot10^{-14}$	$4.21\cdot10^{-14}$	$4.42\cdot10^{-14}$	$1.75\cdot10^{-14}$	$1.2\cdot10^{-14}$
RI	Disease incidences	$2.91\cdot10^{-7}$	$4.65\cdot10^{-7}$	$3.86\cdot10^{-7}$	$3.17\cdot10^{-7}$	$1.85\cdot10^{-7}$
IR	kBq U235 eq.	0.14	0.40	0.34	0.16	0.q0
POF	kBq U235 eq.	$1.14\cdot10^{-2}$	$1.65\cdot10^{-2}$	$1.71\cdot10^{-2}$	$2.81\cdot10^{-2}$	$1.74\cdot10^{-2}$
AC	Mole of H+ eq.	$3.46\cdot10^{-2}$	$4.43\cdot10^{-2}$	$3.61\cdot10^{-2}$	$2.61\cdot10^{-2}$	$1.71\cdot10^{-2}$
EUT	Mole of N eq.	0.11	0.17	0.13	0.12	0.07
EUF	kg P eq.	$2.67\cdot10^{-5}$	$0.82\cdot10^{-4}$	$0.55\cdot10^{-4}$	$3.71\cdot10^{-5}$	$2.32\cdot10^{-5}$
EUM	kg N eq.	$9.95\cdot10^{-3}$	$1.52\cdot10^{-2}$	$1.19\cdot10^{-2}$	$0.96\cdot10^{-2}$	$0.60\cdot10^{-2}$
HTC	CTUh	$1.74\cdot10^{-9}$	$2.57\cdot10^{-9}$	$2.61\cdot10^{-9}$	$1.41\cdot10^{-9}$	$0.65\cdot10^{-9}$
HTNC	CTUh	$8.02\cdot10^{-8}$	$1.33\cdot10^{-7}$	$1.27\cdot10^{-7}$	$0.75\cdot10^{-7}$	$4.80\cdot10^{-8}$
ECFW	CTUe	58.3	113	101	108	67
LU	Pt	2.87	64	92	7.6	4.7
RDM	kg Sb eq.	$4.72\cdot10^{-7}$	$3.16\cdot10^{-4}$	$3.95\cdot10^{-4}$	$3.94\cdot10^{-4}$	$2.42\cdot10^{-4}$
WU	m^3 world equiv.	13.5	5.7	3.7	3.1	1.9
RU	MJ	183	224	226	120	71

Table S4. Environmental impact indicators (EIIs) values for the biochemical route with the contribution of the different processes.

EI	Ud.	Total	Bacteria synthesis	Glucose	Methanol synthesis	Calcium hydroxide	Gypsum stone	Thermal Energy	Ammonia	Process water	Sulphuric acid (96%)	Others
GWP	kg CO2 eq.	12.4	$1.64\cdot10^{-2}$	1.1	$1.37\cdot10^{-2}$	0.53	$1.57\cdot10^{-3}$	9.4	0.15	$5.92\cdot10^{-2}$	0.29	0.76
ODP	kg CFC-11 eq.	$1.21\cdot10^{-14}$	$3.76\cdot10^{-17}$	$5.48\cdot10^{-15}$	$2.68\cdot10^{-16}$	$8.93\cdot10^{-16}$	$2.03\cdot10^{-17}$	$3.38\cdot10^{-16}$	$2.26\cdot10^{-16}$	$1.08\cdot10^{-15}$	$1.29\cdot10^{-15}$	$2.52\cdot10^{-15}$
RI	Disease incidences	$2.91\cdot10^{-7}$	$1.26\cdot10^{-10}$	$1.34\cdot10^{-7}$	$1.65\cdot10^{-10}$	$7.26\cdot10^{-9}$	$9.42\cdot10^{-9}$	$7.31\cdot10^{-8}$	$5.17\cdot10^{-10}$	$1.70\cdot10^{-9}$	$4.58\cdot10^{-8}$	$1.92\cdot10^{-8}$
IR	kBq U235 eq.	0.14	$7.25\cdot10^{-4}$	$7.02\cdot10^{-2}$	$1.42\cdot10^{-4}$	$2.77\cdot10^{-3}$	$6.73\cdot10^{-5}$	$4.07\cdot10^{-3}$	$3.43\cdot10^{-3}$	$1.23\cdot10^{-2}$	$2.56\cdot10^{-2}$	$1.94\cdot10^{-2}$
POF	kBq U235 eq.	$1.14\cdot10^{-2}$	$1.45\cdot10^{-5}$	$1.16\cdot10^{-3}$	$2.00\cdot10^{-5}$	$1.70\cdot10^{-4}$	$1.45\cdot10^{-5}$	$8.42\cdot10^{-3}$	$5.49\cdot10^{-5}$	$1.17\cdot10^{-4}$	$1.06\cdot10^{-3}$	$3.90\cdot10^{-4}$
AC	Mole of H+ eq.	$3.46\cdot10^{-2}$	$1.83\cdot10^{-5}$	$1.85\cdot10^{-2}$	$1.80\cdot10^{-5}$	$1.61\cdot10^{-4}$	$1.11\cdot10^{-5}$	$7.77\cdot10^{-3}$	$5.66\cdot10^{-5}$	$1.42\cdot10^{-4}$	$7.48\cdot10^{-3}$	$4.07\cdot10^{-4}$
EUT	Mole of N eq.	0.11	$3.71\cdot10^{-5}$	$8.06\cdot10^{-2}$	$5.60\cdot10^{-5}$	$7.10\cdot10^{-4}$	$5.86\cdot10^{-5}$	$2.75\cdot10^{-2}$	$1.85\cdot10^{-4}$	$4.86\cdot10^{-4}$	$1.57\cdot10^{-3}$	$1.53\cdot10^{-3}$
EUF	kg P eq.	$2.67\cdot10^{-5}$	$1.02\cdot10^{-8}$	$2.00\cdot10^{-5}$	$1.53\cdot10^{-7}$	$1.54\cdot10^{-7}$	$4.49\cdot10^{-9}$	$1.82\cdot10^{-7}$	$8.07\cdot10^{-8}$	$2.44\cdot10^{-6}$	$8.41\cdot10^{-7}$	$2.84\cdot10^{-6}$
EUM	kg N eq.	$9.95\cdot10^{-3}$	$5.03\cdot10^{-6}$	$6.89\cdot10^{-3}$	$5.13\cdot10^{-6}$	$6.52\cdot10^{-5}$	$5.16\cdot10^{-6}$	$2.48\cdot10^{-3}$	$8.55\cdot10^{-5}$	$5.15\cdot10^{-5}$	$1.43\cdot10^{-4}$	$2.18\cdot10^{-4}$
HTC	CTUh	$1.74\cdot10^{-9}$	$5.56\cdot10^{-12}$	$6.09\cdot10^{-10}$	$4.70\cdot10^{-12}$	$5.96\cdot10^{-11}$	$3.31\cdot10^{-13}$	$7.63\cdot10^{-10}$	$7.95\cdot10^{-12}$	$2.25\cdot10^{-11}$	$1.72\cdot10^{-10}$	$1.01\cdot10^{-10}$
HTNC	CTUh	$8.02\cdot10^{-8}$	$2.31\cdot10^{-10}$	$4.34\cdot10^{-8}$	$1.57\cdot10^{-10}$	$6.48\cdot10^{-9}$	$1.88\cdot10^{-11}$	$1.15\cdot10^{-8}$	$4.00\cdot10^{-10}$	$1.71\cdot10^{-9}$	$7.37\cdot10^{-9}$	$9.00\cdot10^{-9}$
ECFW	CTUe	58.3	0.29	44.0	$1.94\cdot10^{-2}$	0.29	$1.24\cdot10^{-2}$	1.52	0.31	1.12	8.71	2.05
LU	Pt	2.87	$9.56\cdot10^{-3}$	1.23	$2.84\cdot10^{-3}$	0.23	$1.42\cdot10^{-2}$	0.16	$4.62\cdot10^{-2}$	0.27	0.34	0.57
RDM	kg Sb eq.	$4.72\cdot10^{-7}$	$1.36\cdot10^{-9}$	$1.33\cdot10^{-7}$	$3.68\cdot10^{-9}$	$1.35\cdot10^{-8}$	$3.16\cdot10^{-10}$	$2.16\cdot10^{-7}$	$7.24\cdot10^{-9}$	$1.46\cdot10^{-8}$	$4.19\cdot10^{-8}$	$4.07\cdot10^{-8}$
WU	m^3 world equiv.	13.5	$4.05\cdot10^{-4}$	1.59	$6.30\cdot10^{-3}$	$1.02\cdot10^{-2}$	$3.38\cdot10^{-5}$	$2.18\cdot10^{-2}$	$1.67\cdot10^{-3}$	2.16	$3.52\cdot10^{-2}$	9.69
RU	MJ	183	0.45	14.8	0.63	1.59	$2.06\cdot10^{-2}$	144	1.92	0.95	13.0	5.56

Table S5. Environmental impact indicators (EIIs) values for the chemical (Sn-MCM-41) route with the contribution of the different processes.

EI	Ud.	Total	Glucose	Sn-MCM-41	Methanol synthesis	Thermal Eenrgy
GWP	kg CO2 eq.	13.0	1.75	3.26	0.81	7.15
ODP	kg CFC-11 eq.	$4.21{\cdot}10^{-14}$	$0.86{\cdot}10^{-14}$	$2.04{\cdot}10^{-14}$	$1.26{\cdot}10^{-14}$	$2.64{\cdot}10^{-16}$
RI	Disease incidences	$4.65{\cdot}10^{-7}$	$2.10{\cdot}10^{-7}$	$1.73{\cdot}10^{-7}$	$2.75{\cdot}10^{-8}$	$0.55{\cdot}10^{-7}$
IR	kBq U235 eq.	0.40	0.11	0.12	0.16	$3.11{\cdot}10^{-3}$
POF	kBq U235 eq.	$1.65{\cdot}10^{-2}$	$1.81{\cdot}10^{-3}$	$0.58{\cdot}10^{-2}$	$2.65{\cdot}10^{-3}$	$0.64{\cdot}10^{-2}$
AC	Mole of H+ eq.	$4.43{\cdot}10^{-2}$	$2.71{\cdot}10^{-2}$	$0.60{\cdot}10^{-2}$	$3.67{\cdot}10^{-3}$	$0.59{\cdot}10^{-2}$
EUT	Mole of N eq.	0.17	0.12	$1.75{\cdot}10^{-2}$	$0.99{\cdot}10^{-2}$	$2.08{\cdot}10^{-2}$
EUF	kg P eq.	$0.82{\cdot}10^{-4}$	$3.13{\cdot}10^{-5}$	$0.82{\cdot}10^{-5}$	$4.22{\cdot}10^{-5}$	$1.39{\cdot}10^{-7}$
EUM	kg N eq.	$1.52{\cdot}10^{-2}$	$1.08{\cdot}10^{-2}$	$1.68{\cdot}10^{-3}$	$0.9{\cdot}10^{-3}$	$1.88{\cdot}10^{-3}$
HTC	CTUh	$2.57{\cdot}10^{-9}$	$0.96{\cdot}10^{-9}$	$0.82{\cdot}10^{-9}$	$4.08{\cdot}10^{-10}$	$0.58{\cdot}10^{-9}$
HTNC	CTUh	$1.33{\cdot}10^{-7}$	$0.78{\cdot}10^{-7}$	$4.46{\cdot}10^{-8}$	$1.22{\cdot}10^{-8}$	$0.87{\cdot}10^{-8}$
ECFW	CTUe	113	69	29.8	13.6	1.15
LU	Pt	64	1.92	56	5.7	0.12
RDM	kg Sb eq.	$3.16{\cdot}10^{-4}$	$2.08{\cdot}10^{-7}$	$3.16{\cdot}10^{-4}$	$2.13{\cdot}10^{-6}$	$1.64{\cdot}10^{-7}$
WU	m³ world equiv.	5.7	2.5	0.66	2.6	$1.68{\cdot}10^{-2}$
RU	MJ	224	23.2	69	22.2	109

Table S6. Environmental impact indicators (EIIs) values for the chemical (Sn-In-MCM-41) route with the contribution of the different processes.

EI	Ud.	Total	Glucose	Sn-In-MCM-41	Methanol synthesis	Thermal Energy
GWP	kg CO2 eq.	12.6	1.19	5.05	0.40	6.05
ODP	kg CFC-11 eq.	$4.42 \cdot 10^{-14}$	$0.58 \cdot 10^{-15}$	$3.19 \cdot 10^{-14}$	$0.62 \cdot 10^{-14}$	$2.23 \cdot 10^{-16}$
RI	Disease incidences	$3.86 \cdot 10^{-7}$	$1.42 \cdot 10^{-7}$	$1.81 \cdot 10^{-7}$	$1.36 \cdot 10^{-8}$	$4.69 \cdot 10^{-8}$
IR	kBq U235 eq.	0.34	0.07	0.18	0.08	$2.63 \cdot 10^{-3}$
POF	kBq U235 eq.	$1.71 \cdot 10^{-2}$	$1.23 \cdot 10^{-3}$	$0.93 \cdot 10^{-2}$	$1.29 \cdot 10^{-3}$	$0.54 \cdot 10^{-2}$
AC	Mole of H+ eq.	$3.61 \cdot 10^{-2}$	$1.96 \cdot 10^{-2}$	$0.93 \cdot 10^{-2}$	$1.80 \cdot 10^{-3}$	$4.98 \cdot 10^{-3}$
EUT	Mole of N eq.	0.13	0.08	$2.71 \cdot 10^{-2}$	$4.88 \cdot 10^{-3}$	$1.76 \cdot 10^{-2}$
EUF	kg P eq.	$0.55 \cdot 10^{-4}$	$2.12 \cdot 10^{-5}$	$1.26 \cdot 10^{-5}$	$2.07 \cdot 10^{-5}$	$1.17 \cdot 10^{-7}$
EUM	kg N eq.	$1.19 \cdot 10^{-2}$	$0.73 \cdot 10^{-2}$	$2.61 \cdot 10^{-3}$	$4.42 \cdot 10^{-4}$	$1.59 \cdot 10^{-3}$
HTC	CTUh	$2.61 \cdot 10^{-9}$	$0.64 \cdot 10^{-9}$	$1.27 \cdot 10^{-9}$	$2.00 \cdot 10^{-10}$	$4.9 \cdot 10^{-10}$
HTNC	CTUh	$1.27 \cdot 10^{-7}$	$4.60 \cdot 10^{-8}$	$0.70 \cdot 10^{-7}$	$0.59 \cdot 10^{-8}$	$0.73 \cdot 10^{-8}$
ECFW	CTUe	101	46.6	46.6	6.70	0.97
LU	Pt	92	1.30	88	2.78	0.10
RDM	kg Sb eq.	$3.95 \cdot 10^{-4}$	$1.41 \cdot 10^{-7}$	$3.95 \cdot 10^{-4}$	$1.04 \cdot 10^{-6}$	$1.38 \cdot 10^{-7}$
WU	m^3 world equiv.	3.7	1.68	0.94	1.27	$1.42 \cdot 10^{-2}$
RU	MJ	226	15.7	107	10.8	92.5

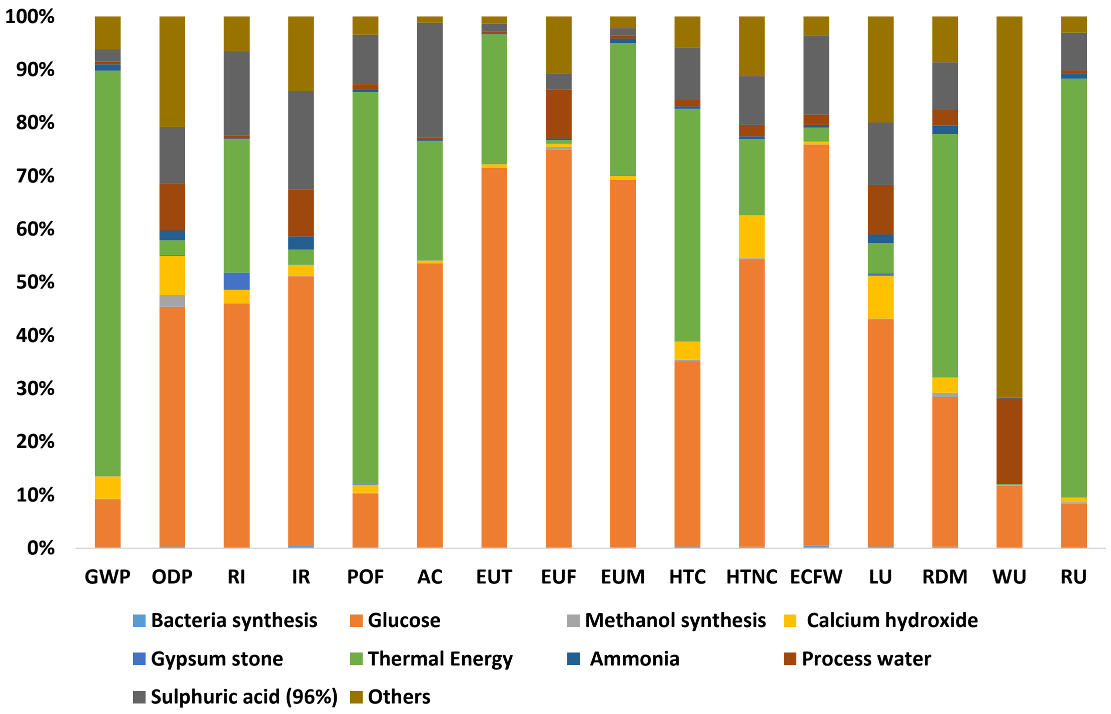

Figure S11. Contribution of the different processes to the environmental impact indicators values for the biochemical route. GWP: global warming potential [kg CO_2 Eq.]; ODP: ozone layer depletion [kg CFC-11 Eq.]; RI: respiratory inorganics [Disease incidences]; IR: ionizing radiation - human health [kBq U-235 Eq.]; POF: photochemical ozone formation - human health [kg NMVOC Eq.]; AC: acidification terrestrial and freshwater [Mole H^+ Eq.]; EUT: eutrophication terrestrial [Mole N Eq.]; EUF: eutrophication freshwater [kg P Eq.]; EUM: eutrophication marine [kg N Eq.]; HTC: human toxicity potential, cancer effects [CTUh]; HTNC: human toxicity potential, non-cancer effects [CTUh]; ECFW: ecotoxicity freshwater [CTUe]; LU: land use [Pt]; RDM: resource use, mineral and metals [kg Sb Eq.]; WU: water use [m^3 world Eq.]; RU: resource use. energy carriers [MJ].

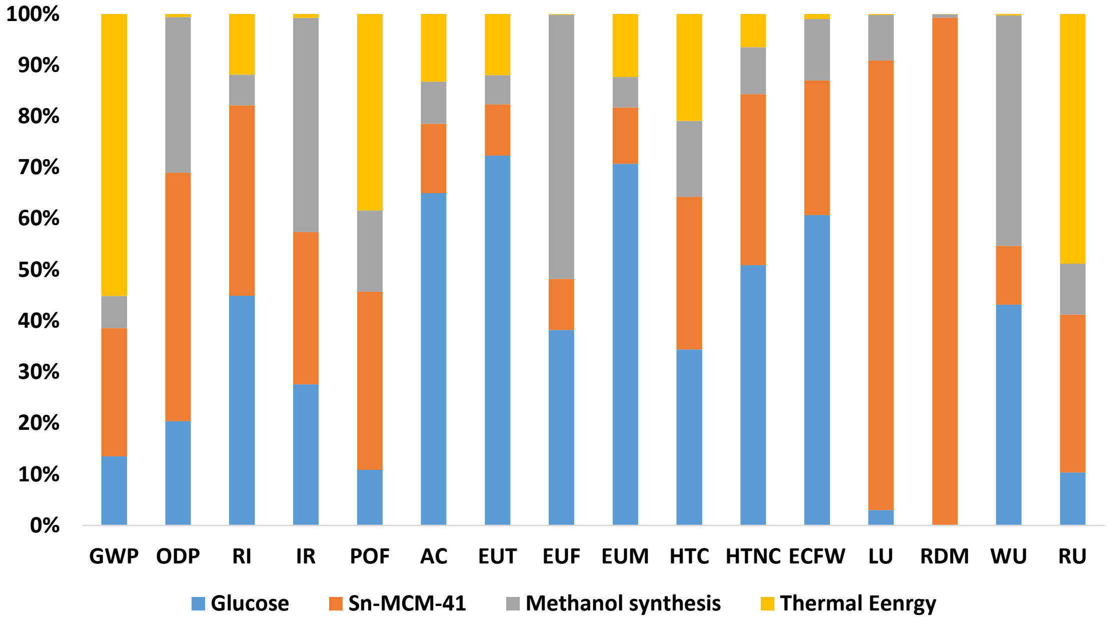

Figure S12. Contribution of the different processes to the environmental impact indicators values for the chemical route (Sn-MCM-41). GWP: global warming potential [kg CO_2 Eq.]; ODP: ozone layer depletion [kg CFC-11 Eq.]; RI: respiratory inorganics [Disease incidences]; IR: ionizing radiation - human health [kBq U-235 Eq.]; POF: photochemical ozone formation - human health [kg NMVOC Eq.]; AC: acidification terrestrial and freshwater [Mole H^+ Eq.]; EUT: eutrophication terrestrial [Mole N Eq.]; EUF: eutrophication freshwater [kg P Eq.]; EUM: eutrophication marine [kg N Eq.]; HTC: human toxicity potential, cancer effects [CTUh]; HTNC: human toxicity potential, non-cancer effects [CTUh]; ECFW: ecotoxicity freshwater [CTUe]; LU: land use [Pt]; RDM: resource use, mineral and metals [kg Sb Eq.]; WU: water use [m^3 world Eq.]; RU: resource use. energy carriers [MJ].

References

(1) Chen. Z.; Shen. Q.; Sun. N.; Wei. W. Life Cycle Assessment of Typical Methanol Production Routes: The Environmental Impacts Analysis and Power Optimization. *J. Clean. Prod.* **2019**. *220* (2019). 408–416. https://doi.org/10.1016/j.jclepro.2019.02.101.

(2) Doran. P. M. *Bioprocess Engineering Principles*; Academic Press. 2012.

(3) Luke. C. Weed Control. *Nurs. Times* **1997**. *93* (33). 40–41. https://doi.org/10.1002/14356007.a28_165.

(4) Huthmacher. K.; Most. D. Cyanuric Acid and Cyanuric Chloride. *Ullmann's Encycl. Ind. Chem.* **2000**. https://doi.org/10.1002/14356007.a08_191.

(5) Simmler. W. Silicon Compounds. Inorganic. *Ullmann's Encycl. Ind. Chem.* **2000**. https://doi.org/10.1002/14356007.a24_001.

(6) Guler. E.; Seyrankaya. A. Extraction of Lead and Silver from Zinc Leach Residue by Brine Leaching. **2010**. No. October.

(7) Fazio. S.; Biganzioli. F.; De Laurentiis. V.; Zampori. L.; Sala. S.; Diaconu. E. *Supporting Information to the Characterisation Factors of Recommended EF Life Cycle Impact Assessment Methods. Version 2. from ILCD to EF 3.0. EUR 29600 EN. European Commission. Ispra. 2018. ISBN 978-92-79-98584-3. Doi:10.2760/002447. PUBSY No. JRC114822.*; 2018.
